# Revealing the Mechanism of Huazhi Rougan Granule in the Treatment of Nonalcoholic Fatty Liver Through Intestinal Flora Based on 16S rRNA, Metagenomic Sequencing and Network Pharmacology

**DOI:** 10.3389/fphar.2022.875700

**Published:** 2022-04-26

**Authors:** Yingying Liu, Yingying Tan, Jiaqi Huang, Chao Wu, Xiaotian Fan, Antony Stalin, Shan Lu, Haojia Wang, Jingyuan Zhang, Fanqin Zhang, Zhishan Wu, Bing Li, Zhihong Huang, Meilin Chen, Guoliang Cheng, Yanfang Mou, Jiarui Wu

**Affiliations:** ^1^ Department of Clinical Chinese Pharmacy, School of Chinese Materia Medica, Beijing University of Chinese Medicine, Beijing, China; ^2^ Institute of Fundamental and Frontier Sciences, University of Electronic Science and Technology of China, Chengdu, China; ^3^ State Key Laboratory of Generic Manufacture Technology of Chinese Traditional Medicine, Linyi, China

**Keywords:** nonalcoholic simple fatty liver, high-fat diet, intestinal flora disorder, 16s sequencing, network pharmacology

## Abstract

**Background:** The incidence of Nonalcoholic Fatty Liver (NAFL) is increasing year by year, growing evidence suggests that the intestinal flora plays a causative role in NAFL. Huazhi Rougan Granule (HRG) is commonly used in the clinical treatment of NAFL. It is reported that it can reduce lipids and protect the liver, but no research has confirmed whether the drug’s effect is related to the intestinal flora. Therefore, we investigated whether the effect of HRG is related to the regulation of intestinal flora to further explore the mechanism of HRG in the treatment of NAFL through intestinal flora.

**Methods:** In this study, C57BL/6J mice were fed a high-fat diet for 10 weeks, and the high-fat diet plus HRG or polyene phosphatidylcholine capsules were each administered by gavage for 5 weeks. High-throughput sequencing, network pharmacology, and molecular docking were used to explore the mechanism of HRG in the treatment of NAFL through intestinal flora.

**Results:** HRG treatment can reduce body weight gain, lipid accumulation in liver and lipogenesis and reduce serum biochemical indexes in high-fat-fed mice. Analysis of intestinal flora showed that HRG changed the composition of intestinal flora, which was characterized by a decrease in the Firmicutes/Bacteroidetes ratio. Moreover, the species distribution was significantly correlated with AKP, HDL-C, and TG. Metagenetic analysis showed that HRG altered the functional composition and functional diversity of microorganisms, which was mainly characterized by an increase in the abundance of metabolic pathways. The network pharmacology results show that the mechanism of HRG in the treatment of NAFL through intestinal flora is mainly reflected in the biological process of gene function and related to infectious diseases, immune systems, and signal transduction pathways, such as cytokine-cytokine receptor interaction, Chagas disease, IL-17 signaling pathway and other signaling pathways.

**Conclusion:** These results strongly suggest that HRG may alleviate NAFL by preventing IFD.

## Graphical Abstract

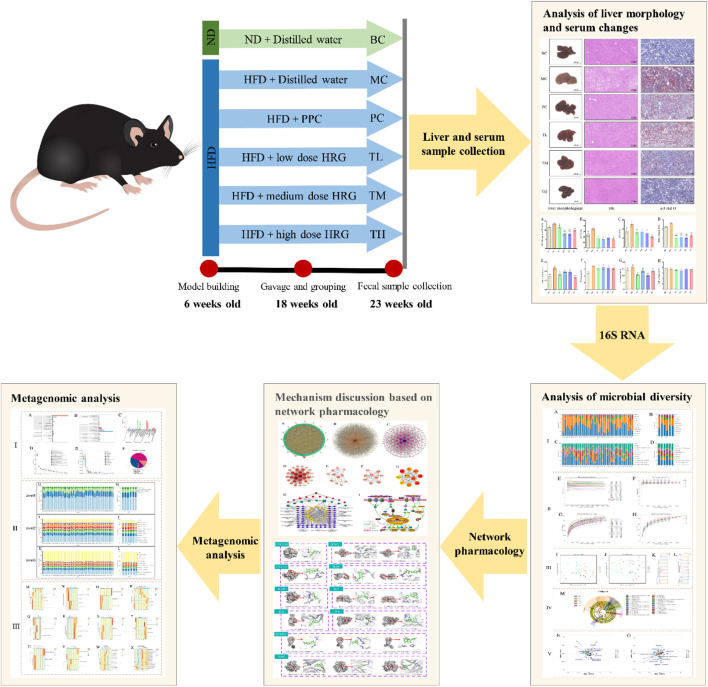

## Background

Nonalcoholic Fatty Liver (NAFL) is a common, multifactorial, and less significant liver disease whose incidence is increasing worldwide. The occurrence of NAFL is mainly related to unhealthy dietary habits and lifestyles. This leads to pathological accumulation of fat droplets in hepatocytes (Cobbina and Akhlaghi, 2017; Romero-Gómez et al., 2017). There is increasing evidence that obesity, cardiovascular disease, and type 2 diabetes are closely related to the progression of NAFL and represent an increasing burden to society (Eckel et al., 2010; Yang et al., 2015; Graffy and Pickhardt, 2016; Younossi et al., 2019; de Vries et al., 2020; Deprince et al., 2020). Although NAFL is usually clinically asymptomatic, it can progress over time to non-alcoholic steatohepatitis, cirrhosis, and end-stage liver disease (Papatheodoridi and Cholongitas, 2018; Zhou et al., 2020). Currently, lifestyle changes are the mainstay of treatment, including dietary changes and exercise (Younossi et al., 2018; Kořínková et al., 2020). Therefore, the development of drugs and nutraceuticals for NAFL remains a challenge for all scientists (Targher et al., 2020).

In recent years, intestinal flora has gradually attracted the attention of scientists. A growing number of studies show that intestinal flora is closely related to human health homeostasis, which opens a new direction for us to understand the occurrence and progression of NAFL (Milani et al., 2017; Mohajeri et al., 2018; Gomaa, 2020; Kim et al., 2020; Manor et al., 2020). Studies have shown that intestinal flora can affect lipid metabolism and lipid levels in blood and tissues in mice and humans (Zhou et al., 2017; Wang et al., 2018; Kong et al., 2019; Yuan et al., 2019; Hong et al., 2020; Yiu et al., 2020). In addition, animal and clinical studies have shown that regulation of intestinal flora and its metabolites can influence the degree of a high-fat diet-induced hepatic steatosis in NAFL mice and NAFL patients, thereby interfering with the occurrence and development of NAFL (Jiang et al., 2015; Moreira et al., 2018; Wu et al., 2019; Nakano et al., 2020; Zhang et al., 2021). Therefore, there are good reasons to believe that the composition of intestinal flora may help predict the severity of NAFL and may be a new therapeutic target for NAFL.

Huazhi Rougan Granule (HRG) is widely used to treat dampness-heat obstruction of NAFL. It has the function of clearing heat and detoxifying, eliminating blood stasis, and softening the liver. The composition of HRG is Yinchen (YC), Juemingzi (JMZ), Dahuang (DH), Zexie (ZX), Zhuling (ZL), Shanzha (SZ), Cangzhu (CZ), Baizhu (BZ), Chenpi (CP), Gualou (GL), Nvzhenzi (NZZ), Mohanlian (MHL), Gouqizi (GQZ), Xiaoji (XJ), Chaihu (CH), Gancao (GC). Studies have shown that this formula can not only improve lipid deposition in the liver, protect liver cell membranes and reduce liver damage but also protect the intestinal barrier and regulate intestinal flora (Niu et al., 2011; Chen et al., 2015; Gao et al., 2021; Zhang et al., 2021; Zhang et al., 2021).

Since intestinal flora disorder is the breakthrough point in network pharmacology, the treatment of NAFL-related intestinal flora disorder by HRG was investigated to explore the mechanism of HRG in the treatment of NAFL by intestinal flora in multiple dimensions. Therefore, this study not only explored the mechanism of HRG in the treatment of NAFL by intestinal flora using network pharmacology but also established a NAFL model for experiments. The intervention effect in NAFL mice was analyzed by the high-throughput sequencing method to analyze the diversity of intestinal flora in mice to provide a better basis for further exploration of the mechanism of HRG in the treatment of NAFL through intestinal flora.

## Materials and Methods

### Experimental Animals

Seventy-two 6-week-old SPF-grade male C57BL/6J mice, weighing between 20–22 g, were purchased from Beijing Speifu Biotechnology Co., Ltd. They were housed in the ICV cage of the Experimental Animal Center of Beijing University of Traditional Chinese Medicine (*n* = 5/cage). Animals were housed at 20 ± 2°C in a 12-h light/12-h dark cycle and had free access to food and water.

### Main Reagents in the Experiment

Reagents: Huazhi Rougan Granule (Lunan Pharmaceutical Co., Ltd.); Polyene Phosphatidylcholine Capsule (Yishanfu); high-fat diet [78.8% basic feed +10% lard +10% egg yolk powder +1% cholesterol +0.2% sodium cholate, product number is SCXK (Jing) 2019-0010, Beijing Speifu Biotechnology Co., Ltd.]; normal diet [corn, soybean meal, fish meal, flour, bran, salt calcium ammonium phosphate, stone powder, a variety of vitamins, a variety of trace elements, amino acids, etc, product number is SCXK (Jing) 2019-0010, Beijing Speifu Biotechnology Co., Ltd.]; 4% tissue cell fixative (Beijing Bairuiji Biotechnology Co., Ltd.); AKP, ALT, AST, TG, TC, HDLC, LDLC assay kit (Nanjing Jiancheng).

### Animal Grouping and Model Establishment

After 1 week of adaptive feeding of male C57 mice, 12 mice were randomly selected as a blank control group (BC) and fed with a normal diet (ND); the remaining mice were fed with high-fat diet (HFD) to replicate the NAFL model. After 10 consecutive weeks, relevant indicators were evaluated. Mice that passed the evaluation could be considered successful modeling, gavage at week 12, and sampled at week 17. Gavage continued for 5 weeks. The successfully established mice were randomly divided into a model control group (MC), a positive drug control group (PC), and a high-medium-low-dose HRG group (TH/TM/TL), with 10 mice in each group. Finally, there were nine mice in each group after sampling. HRG and Polyene Phosphatidylcholine Capsule (PPC) were administered at equivalent doses converted from the upper clinical limits of the human body, with doses of 3.12 g/kg and 0.18 g/kg respectively. The high, medium and low doses of HRG were 4, 2, and 1 times, respectively, the equivalent dose after conversion.

### Collection and Preparation of Samples

After collection of mouse feces, the feces were placed in sterile cryopreservation tubes, quickly frozen in liquid nitrogen, and stored in an ultra-low temperature refrigerator at −80°C for subsequent analysis of intestinal flora diversity. After the mice fasted for 12 h, the eyeballs were removed, and the blood was collected in a 2 ml sterile centrifuge tube, centrifuged at 3,000 r/min for 15 min at 4°C. The supernatant was stored in a centrifuge tube for subsequent biochemical index analysis. One-third of mouse liver lobes were fixed in 4% paraformaldehyde fixative, routinely processed, embedded in paraffin, 3 μm sections were stained with hematoxylin and eosin (HE) for histological analysis, and frozen sections (8 μm) were stained with oil red O. The fat accumulation and inflammatory response of liver tissue were observed under the microscope.

### 16S rRNA and Metagenomic Sequencing

16S rRNA sequencing: After extraction of the total DNA from the sample, all primers were designed according to the conserved region. 338F (5′- ACT​CCT​ACG​GGA​GGC​AGC​A-3′) and 806R (5′- GGACTACHVGGGTWTCTAAT-3′) were used for PCR amplification of the V3-V4 region of the 16S rDNA gene to perform the Illumina deep sequencing. PCR products were detected by 1.8% agarose gel electrophoresis. Metagenome sequencing: extract DNA from fecal samples, detect the DNA of the sample, and fragment the DNA with ultrasonic waves after passing the test. Then, the fragmented DNA is purified, the end repaired, A is added to the three ends, and the sequencing adapter is ligated. Fragment size selection was performed by agarose gel electrophoresis, and PCR amplification was performed to form a sequencing library. The constructed libraries are first checked for quality, and qualified libraries are sequenced on the Illumina sequencing platform. Beijing BioMarker Technologies Co., Ltd. provided library construction and sequencing Beijing BioMarker Technologies Co., Ltd. (Beijing, China).

### Network Pharmacology Analysis

Through the Traditional Chinese Medicine Systems Pharmacology Platform (TCMSP, https://tcmspw.com/tcmsp.php) (Ru et al., 2014; Zhang et al., 2020) and the Integrative Pharmacology-based Research Platform of Traditional Chinese Medicine (TCMIP v2.0, http://www.tcmip.cn/TCMIP/index.php/Home/) (Wang et al., 2021), the active ingredients of HRG were screened. The TCMSP database and the Swiss Target Prediction platform (http://www.swisstargetprediction.ch/) (Daina et al., 2019) were used to identify the targets of the main active ingredients. The Uniprot database (https://www.uniprot.org/) (UniProt Consortium, 2021) is used to find the gene information corresponding to the targets. After integration, the active components and related target information of HRG were obtained. The Gene Cards database (https://www.genecards.org) (Stelzer et al., 2016), DigSee database (http://210.107.182.61/geneSearch/) (Kim et al., 2013) and NCBI Gene (https://www.ncbi.nlm.nih.gov/gene/) (NCBI Resource Coordinators, 2018) were used to search for targets related to NAFL and IFD. The above targets were respectively integrated and duplicate genes were removed. A total of 198 targets related to IFD and 1,395 targets related to NAFL were obtained.

Herb-compound-target network, PPI network and multi-element network of “herb-key compound-key target-disease-KEGG pathway” of HRG were constructed by Cytoscape 3.7.2. (Otasek et al., 2019). Comprehensive modular analysis and cytoHubba analysis were used to screen the key targets of HRG in the treatment of NAFL through the intestinal flora. GO enrichment analysis and KEGG pathway enrichment analysis were performed on the targets in the intersection network using R 4.0.3 (Layeghifard et al., 2018; The Gene Ontology Consortium, 2019; Kanehisa and Sato, 2020). The potential active ingredients obtained by the analysis were docked onto the potential targets. Autodock Tools 1.5.6 was used to perform preprocessing such as water removal, hydrogenation, and atom typesetting for proteins and small molecule compounds, Autodock Vina 1.1.2 was used to perform molecular docking calculations, and Pymol 2.3.2 (https://pymol.org/2/) was used to visualize the result (Trott and Olson, 2010; Mooers, 2020; Eberhardt et al., 2021).

### Bioinformatics Analysis

Microbial diversity analysis: FLASH v1.2.11 was used to stitch the original data. The spliced sequences were quality filtered and chimeras were removed to obtain high-quality Tags sequences. Sequences were clustered at a similarity level of 97%, and OTUs were filtered with a threshold of 0.005% of all sequenced sequences. Alpha diversity and beta diversity were analyzed using the BMK Cloud platform (www.biocloud.ent).

Metagenome analysis: use of Trimmomatic (version 0.33) software to filter raw data (Raw Tags); use of bowtie2 (version 2.2.4) to align with host genome sequence to remove host contamination; MEGAHIT (Version 1.1.2) was used for metagenomic assembly to filter contig sequences shorter than 300 bp; MetaGeneMark (Version 3.26) was used for gene prediction; cd-hit (Version 4.6.6) was used to remove redundancy. The similarity threshold was set a 95%, and the coverage threshold was set at 90%. Both functional diversity analysis and species diversity analysis are analyzed on the BMK Cloud platform (www.biocloud.ent).

### Statistical Analysis

The relevant results are expressed as mean ± SE. SPSS software was used for data analysis and GraphPad Prism was used for visualization. *p* values of less than 0.05 were considered statistically significant.

## Results

### Lipid Accumulation in Mice Induced by HFD

**FIGURE 1:**
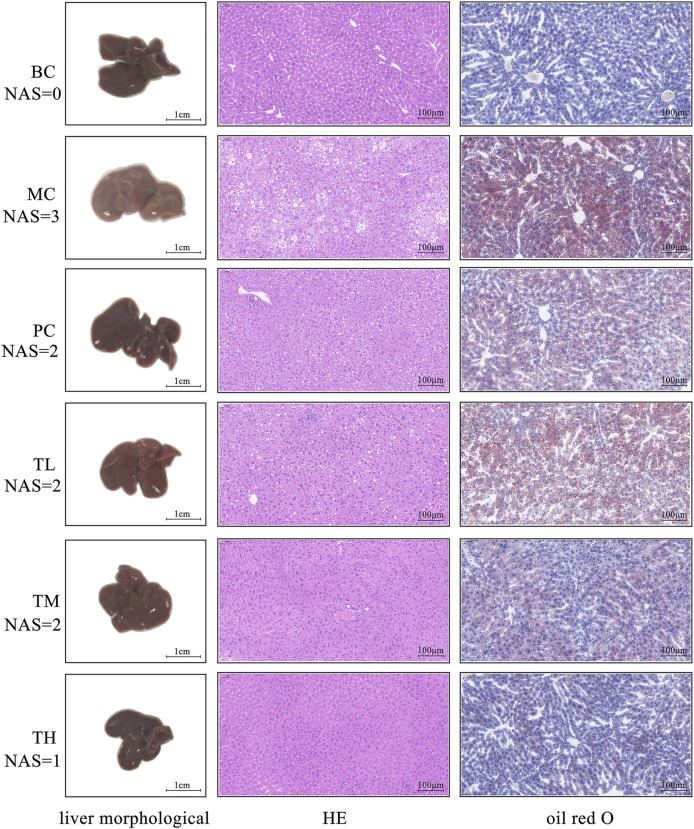
Effects of HRG on liver morphology, liver histopathology (HE staining, ×20) and fatty lesions in mice (oil red O staining, ×20).

As shown in Figure 1, in the model group compared with the BC group, the fat accumulation in the liver increased, the liver volume increased, white color, and the edge became blunt. After administration, the degree of redness of the liver improved in each group, especially in the group TH. The results of HE and oil red O staining showed more balloon denatured hepatocytes and more red lipids in the liver tissue of the model group but less in the administration group. The pathological degree of the MC group was the most severe, that of the TH group was the mildest, and there was no significant difference between the TL group and the PC group. Therefore, HRG ameliorates HFD-induced hepatic fat accumulation and weight gain, and HRG may improve liver lesions in a dose-dependent manner.

### Serum Analysis

Compared with the blank group, the indexes of liver enzymes and blood lipids were higher in the model group, among which there were significant differences in TC, HDL-C, LDL-C, AKP, ALT, and AST. However, there was no significant difference in TG. After administration, liver enzymes and blood lipids were lower than in the model group. There were significant differences in ALT, AST and HDL-C between the MC and administration groups. There were no significant differences in TC, ALT and HDL-C between the administration groups. There were differences in AKP, AST, TG and LDL-C between the HRG group and the PC group.

**FIGURE 2:**
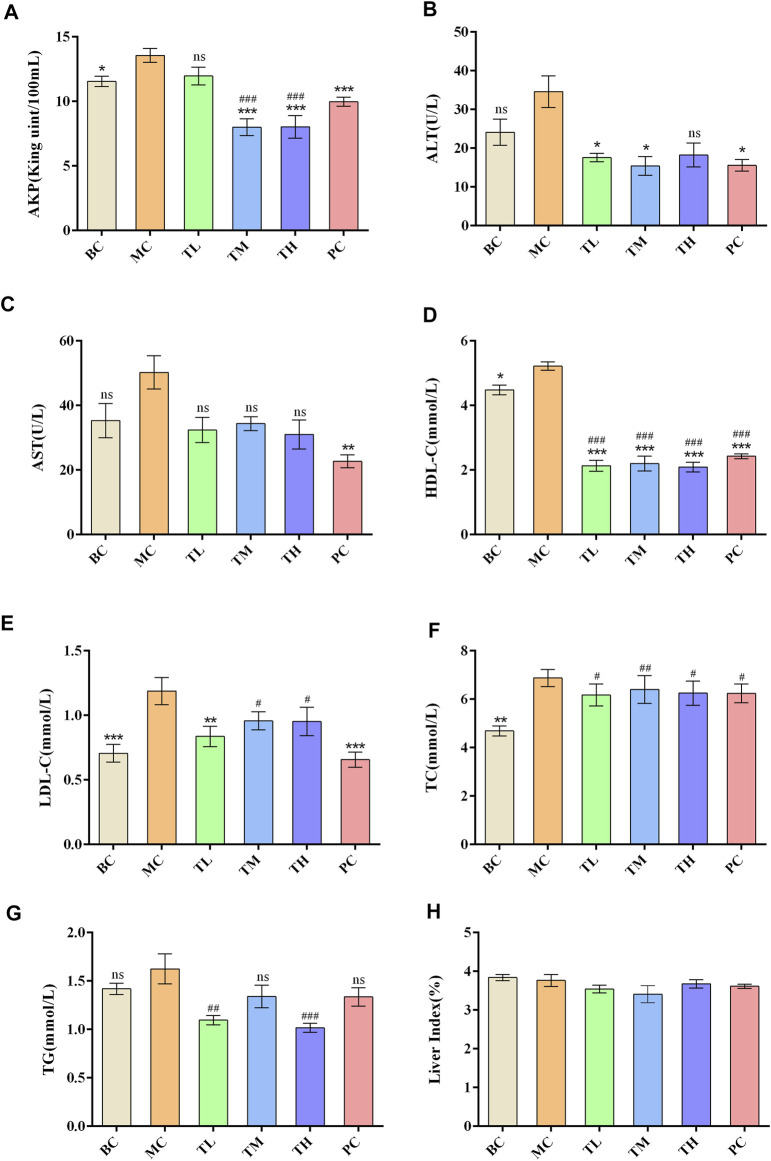
Effect of HRG on biochemical indexes and statistical histogram of organ index in mice. **(A)** Effects of HRG on serum AKP level in mice. **(B)** Effects of HRG on serum ALT level in mice. **(C)** Effects of HRG on serum AST level in mice. **(D)** Effects of HRG on serum HDL-C level in mice. **(E)** Effects of HRG on serum LDL-C level in mice. **(F)** Effects of HRG on serum TC level in mice. **(G)** Effects of HRG on serum TG level in mice. **(H)** Effects of HRG on liver index in mice. Compared with the model group, *p < 0.05, **p < 0.01, ***p < 0.001; compared with the blank group, #*p* < 0.05, ##*p* < 0.01, ###*p* < 0.001).

The effect of reducing AKP in the TM and TH group was better than in the TL group, and the effect of lowering in the TG group was better than in the TM group. The effect of lowering AKP in the PC group was better than in the TL group but worse than in the TM and TH groups. In addition, the effect of LDL-C lowering in the PC group was better than in the TM and TH groups. The above differences were statistically significant. This suggest that HFD can lead to abnormal metabolism of blood lipids and liver enzymes, and that HRG can improve this. Compared with positive drugs, HRG has advantages in improving HDL-C, TG, and AKP, and the degree of improvement may be dose-dependent. There was no significant difference in organ index between groups. The comparison of the different indexes of each group is shown in Table 1, and the effects of HRG on the biochemical indexes and organ index of mice are shown in Figure 2.

**TABLE 1:** Comparison of serum biochemical indexes of mice in each group after 5 weeks of gavage (‾x±se, *n* = 9).

Group	HDLC (mmol/L)	LDLC (mmol/L)	TG (mmol/L)	TC (mmol/L)	AST (U/L)	ALT (U/L)	AKP(King unit/100 ml)
BC	4.48 ± 0.15^*^	0.71 ± 0.07^****^	1.41 ± 0.06	4.69 ± 0.21^***^	35.31 ± 5.27	24.11 ± 3.40	11.55 ± 0.39^*^
MC	5.22 ± 0.13	1.19 ± 0.11	1.62 ± 0.15	6.87 ± 0.35	50.25 ± 5.15	34.61 ± 4.08	13.56 ± 0.53^#^
TL	2.13 ± 0.17^**,##^	0.84 ± 0.08^**^	1.10 ± 0.05^*,##,▲^	6.17 ± 0.45^##^	32.38 ± 3.91	17.57 ± 1.06^*^	11.96 ± 0.70^▲^
TM	2.20 ± 0.23^**,##^	0.96 ± 0.07^#,▲^	1.34 ± 0.12	6.40 ± 0.57^##^	34.35 ± 2.19^▲^	15.39 ± 2.43^*^	7.80 ± 0.65^***,###,▲^
TH	2.10 ± 0.15^**,##^	0.95 ± 0.11^▲^	1.02 ± 0.05^##,*,▲^	6.24 ± 0.50^##^	31.02 ± 4.48	18.25 ± 3.07	8.03 ± 0.87^***,###,▲^
PC	2.43 ± 0.08^**,##^	0.66 ± 0.06^**^	1.34 ± 0.10	6.23 ± 0.39^##^	22.67 ± 1.99^**^	15.58 ± 1.49^*^	9.97 ± 0.34^***^

Compared with the model group, *p < *0.05*, **p < *0.01*, ***p < *0.001*.

Compared with the blank group, #*p* < *0.05*, ##*p* < *0.01*, ###*p* < *0.001*.

Compared with the PC, group, ▲*p* < *0.05*, ▲▲*p* < *0.01*, ▲▲▲*p* < *0.001*.

### Microbial Diversity Analysis

The intestinal flora is considered to play a causal role in the pathogenesis of NAFL. We evaluated the effects of administration on intestinal flora composition by high-throughput sequencing of the bacterial 16S rRNA V3 + V4 region. High-throughput sequencing generated a total of 4,191,545 raw reads from 54 samples. After screening, a total of 4,178,702 high-quality reads were obtained. Based on a similarity level of 97%, all effective reads were clustered into OTUs for OTU cluster analysis and species taxonomy analysis. The quality evaluation of the sequencing data is shown in Supplementary Table S1.

**FIGURE 3:**
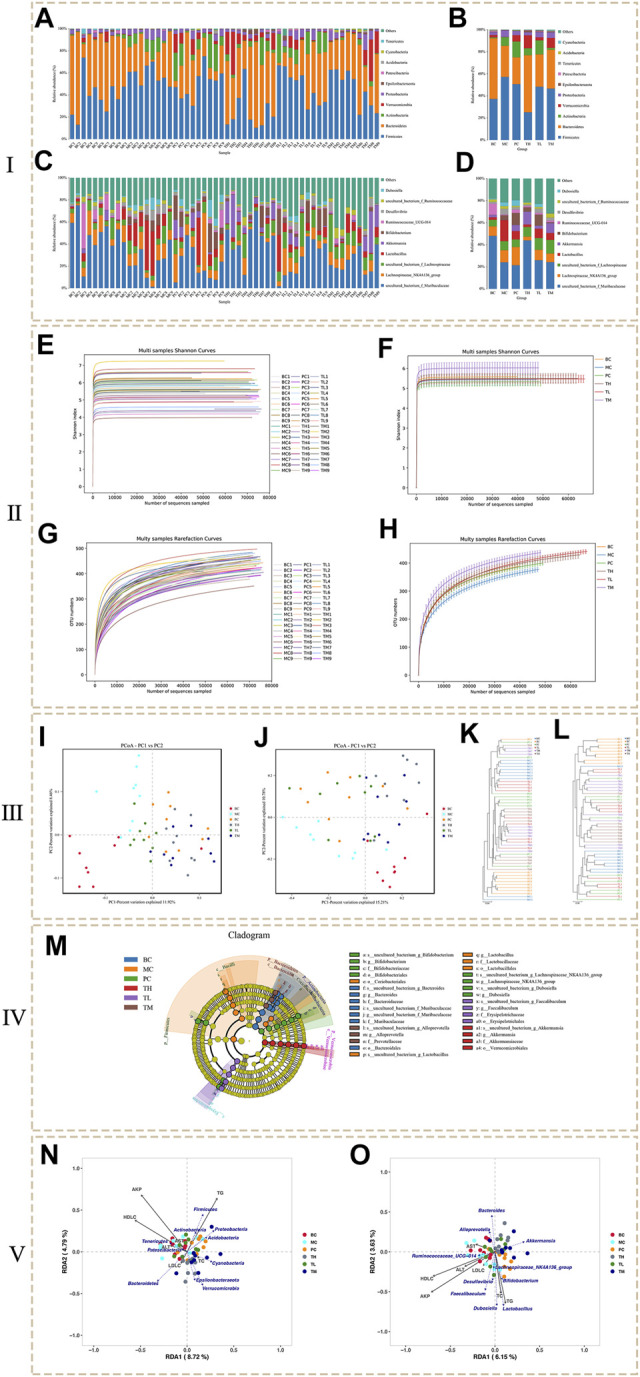
The results of microbial diversity analysis. (Ⅰ: Histogram of species distribution. Ⅱ: Dilution curve and Shannon curve. Ⅲ: Beta analysis based on OTU. Ⅳ: LEfSe analysis. Ⅴ: RDA analysis.)

In this study, the flora was identified and analyzed at the phylum, class, order, family, and genus levels. Finally, a total of 17 phyla, 26 classes, 56 orders, 86 families and 187 genera were identified. The figure shows the annotation of species and the taxonomic analysis. The histogram of species distribution (Figures 3A–D) shows that species are mainly distributed in Firmicutes and Bacteroidetes, followed by Actinobacteria, Verrucomicrobia and Proteobacteria. In addition, is they are mainly distributed in the genera of uncultured_bacterium_f_Muribaculacea and lachnospiraceae NK4A136 group, followed by uncultured_bacterium_f_Lachnospirae, *Lactobacillus* and Akkermansia.

At the phylum level (Figures 3A,B), the levels of Firmicutes, Proteobacteria and Actinobacteria were higher in the MC group than in the BC group, whereas the levels of Bacteroidetes and Verrucomicrobia were lower. Compared to the BC group, the levels of Firmicutes and Proteobacteria decreased in the administration group, especially in the TH group; Actinobacteria decreased in the TH and TM groups, most significantly in the TM group, but increased in the PC and TL groups. Bacteroidetes were significantly higher in the TH and TM groups than in the MC group, and Verrucomicrobia increased most significantly in the TH and TM groups. Compared with the BC group, the proportion of *Lactobacillus* and Desulfovibrio was higher in the MC group at the genus level (Figures 3C,D). In comparison, Akkermansia and Ruminococcaceae UGG-014 accounted for a lower proportion. After administration, *Lactobacillus* (decreased the most in TH group) and Desulfovibrio (decreased the most in TH group) decreased, Akkermansia (increased the most in TH group) and ruminococcaceae UGG-014 (increased the most in PC group) increased. After administration, *Lactobacillus* and Desulfovibrio decreased and Akkermansia increased, especially in the TH group; Ruminococcaceae UGG-014 increased, especially in the PC group. Therefore, it is tentatively suggested that HRG can increase Bacteroidetes, Verrucomicrobia, uncultured bacterium f Lachnospiceae and Akkermansia, and reduce Firmicutes and Proteobacteria, *Lactobacillus* and Desulfovibrio. HRG can improve NAFL by changing the structure of intestinal flora of NAFL mice.

The analysis of alpha diversity can be represented by the index in Table 2. The larger the index, the higher the diversity of the sample. The results showed no significant difference between the Shannon index and the Simpson index in the community diversity evaluation. The ACE index and the Chao index in community richness evaluation showed that the richness of the TL and TM groups was significantly higher than that of the BC group, the MC group and the PC group. Shannon index from large to small TM > BC > TH > TL > MC > PC, Simpson index from large to small TM > BC > TL > TH > MC > PC, ACE index from large to small TM > TL > TH > BC > PC > MC, PD whole tree index from large to small TM > TL > TH > PC > BC > MC, Chao1 index from large to small TM > TL > TH > BC > PC > MC. Various indexes of alpha diversity showed that HRG could improve the diversity of NAFL mice, and the curative effect was better than that of the PC group. At the same time, the coverage rate of each group of samples is greater than 99%, indicating that the probability of undetected sequences in the samples is very low, and the sequencing depth can accurately reflect the composition diversity of the flora. The dilution curve shows that when the curve becomes gradually flat, increasing the sequencing depth has no significant impact on alpha diversity, as shown in Figures 3E,F. The Shannon index curve shows that when the curve tends to become flat, the characteristic species do not increase with the increase of sequencing quantity, as shown in Figures 3G,H.

**TABLE 2:** Alpha diversity statistics (‾x±se, *n* = 9).

Group	BC	MC	PC	TH	TL	TM
Shannon index	5.5847 ± 0.1425	5.4356 ± 0.2673	5.3317 ± 0.2126	5.4967 ± 0.2489	5.4752 ± 0.187	6.0296 ± 0.2885^▲^
Simpson index	0.9445 ± 0.0089	0.9311 ± 0.0208	0.9228 ± 0.0157	0.9346 ± 0.0158	0.9412 ± 0.0115	0.9509 ± 0.0147
ACE index	464.1403 ± 7.6454	452.5716 ± 6.7194	457.6025 ± 5.263	472.0735 ± 9.1531	492.6638 ± 7.061^**,#,▲▲^	496.8799 ± 9.3045^***,##,▲▲^
Chao1 index	472.0195 ± 10.5494	457.5262 ± 8.1369	464.9995 ± 5.4769	477.7676 ± 9.7134	492.5886 ± 7.293^**,▲^	505.3993 ± 9.6354^***,##,▲▲^
PD whole tree index	25.1176 ± 0.3986	24.8614 ± 0.3622	25.3351 ± 0.2404	26.193 ± 0.4081^*,#^	26.721 ± 0.3868^**,##,▲▲^	26.9273 ± 0.2881^***,##,▲▲^
OTU	414.56 ± 8.056	402.89 ± 9.137	420.11 ± 5.397	434.44 ± 11.973^*^	446.22 ± 7.240^**,#,▲^	457.11 ± 8.389^***,##,▲▲^
Coverage (%)	99.8878 ± 0.0128	99.9056 ± 0.0112	99.9167 ± 0.0058^#^	99.9167 ± 0.0071^#^	99.9078 ± 0.0040	99.9111 ± 0.0102

Compared with the model group, *p < *0.05*, **p < *0.01*, ***p < *0.001*.

Compared with the blank group, #*p* < *0.05,* ##*p* < *0.01*, ###*p* < *0.001*.

Compared with the PC, group, ▲*p* < *0.05*, ▲▲*p* < *0.01*, ▲▲▲*p* < *0.001*.

The comparison of the changing trend of β diversity of intestinal flora in each group is based on visual analysis of ecological differences by the Jaccard and Bray-Curtis PCoA algorithm based on independent OTU. As shown in the Jaccard PCoA in Figure 3I, the contribution of principal coordinates 1 (PC1) and PC2 to the distribution of samples is 11.92 and 8.46%, respectively. The clustering of the intestinal flora shows that there are obvious differences in the microbial community between the samples of BC and MC groups, and there are obvious differences in the microbial community between the administration group, BC group and MC group. In addition, the Bray-Curtis PCoA confirms these findings (Figure 3J). PC1 (15.21%) can better distinguish the microecology of BC and MC group, MC and TM, TH group. When PC2 (10.78%) was introduced, PC and BC, PC and MC, TH and BC, and TH and MC could also be significantly separated (Figure 3J). Community similarity analysis is another method that uses hierarchical clustering of the distance matrix to represent β diversity. The similarity and differences between samples can be described by the dendritic structure (Figures 3K,L). These data further indicated that the BC and MC groups had large differences in microbial community structure and that the HRG administration group and the control group had large differences in microbial community structure.

In order to find the biomarker flora with statistical abundance differences between the different groups, LEfSe analysis of the samples between the groups was performed. Figure 3M shows the evolutionary branch diagram of LEfSe analysis. The results showed that Bacteroidetes, Actinobacteria, Firmicutes, and Verrucomicrobia were significantly different species at the phylum level. At the genus level, Bifidobacterium, *Bacteroides*, Alloprevotella, *Lactobacillus*, Lachnospiraceae_ NK4A136_ group, Dubosiella, Faecalibaculum and Akkermansiaceae were significantly different species. *t*-test at the phylum and genus levels between the groups showed (Supplementary Tables S2, S3) that the dominant bacterial groups of NAFL mice and HRG group were mainly Firmicutes, Proteobacteria, Actinobacteria, and Bacteroidetes. At the genus levels, they were mainly Adlercreutzia and Ruminococcaceae_ UCG-013, Sphingomonas, etc. It is suggested that HRG may improve NAFL by changing the flora of Firmicutes, Proteobacteria, Actinobacteria and Bacteroidetes.

RDA analysis was performed for intestinal flora abundance and serum index parameters (Figure 3N: phylum level, Figure 3O: genus level). The results showed that AKP, HDLC, TC, and TG were highly correlated with species distribution, and they were distributed in the same direction. At the genus level, they were positively correlated with Faecalibaculum, Desulfovibrio, Dubosiella and *Lactobacillus*, and negatively correlated with *Bacteroides*, Akkermansia and Alloprevotella. At the phylum level, AKP, HDL-C and TG were highly correlated with species distribution, positively correlated with Firmicutes and Actinobacteria, and negatively correlated with Epsilonbacteraeota and Verrucomicrobia. Acidobacteria and Proteobacteria were negatively correlated with AKP and HDL-C and positively correlated with TG. Bacteroidetes were positively correlated with HDL-C and negatively correlated with AKP and TG. The results showed that these changes in colony abundance were closely related to the structural changes of intestinal flora in NAFL mice before and after administration.

### Network Pharmacology

**FIGURE 4:**
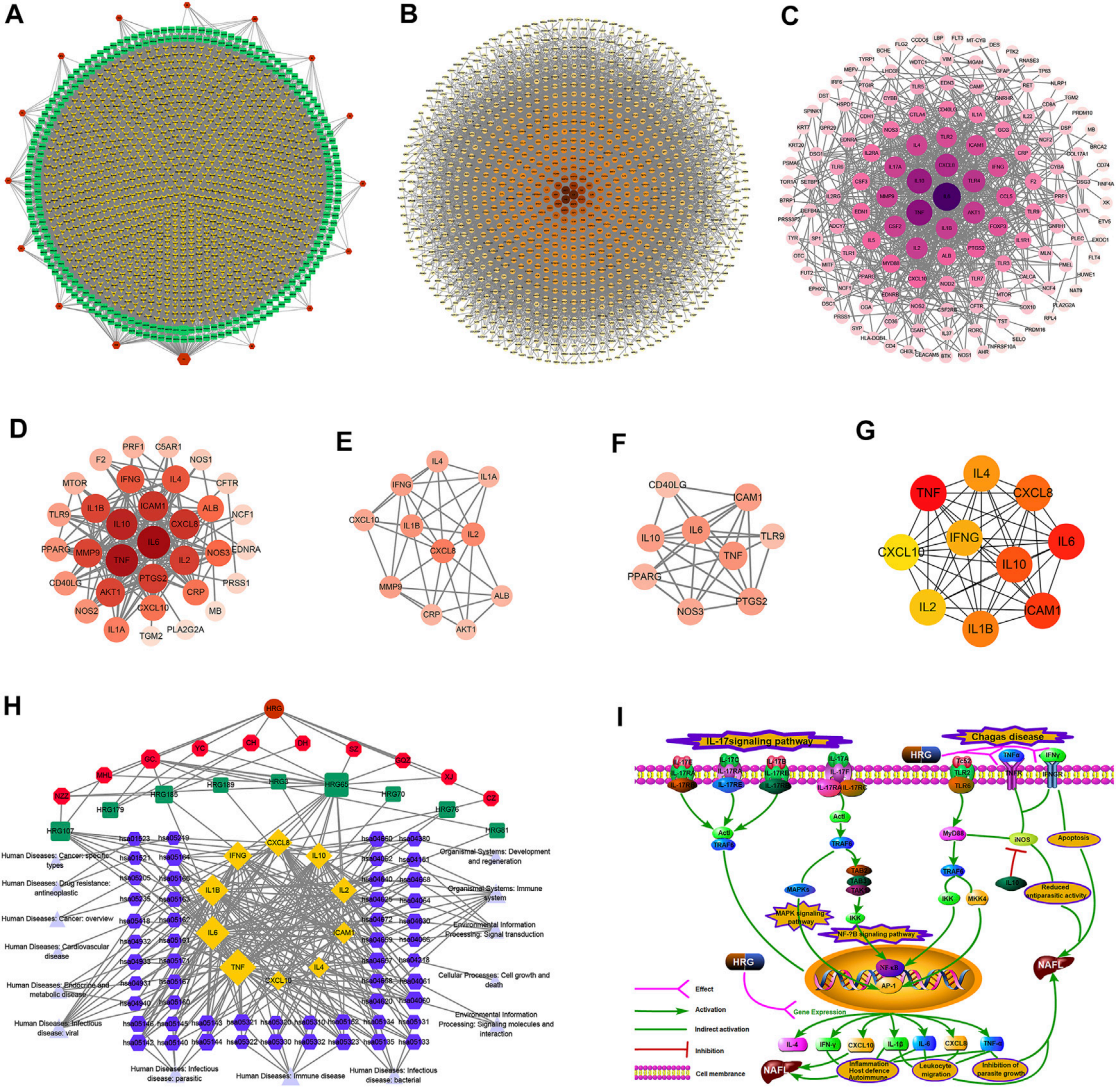
Network construction and correlation analysis. [**(A)** Herb-compound-target network of HRG. **(B)** PPI network related to NAFL. **(C)** PPI network related to IFD. **(D)** PPI network of HRG-IFD-NAFL merge targets. **(E)** Module 1. **(F)** Module 2. **(G)** Hub gene. **(H)** Herb-key compound-potential target-pathway network. **(I)** Illustration of crucial putative biological progress caused by key targets].

249 effective compounds were obtained by screening and removing the repetitive compounds among the herbs. The above compounds were used for target prediction, correction, and deletion of duplicate targets, and a total of 1,186 standard gene names for the targets were obtained. The “herb-compound-target” network of HRG is shown in Figure 4A (the yellow nodes represent drug targets, the green nodes represent compounds, and the red nodes represent herb), and 14 compounds in the 20 nodes with the highest degree value were selected as key compounds (Table 3). The PPI network of NAFL is shown in Figure 4B and the PPI network of IFD is shown in Figure 4C. The targets related to NAFL, the targets related to IFD, and the targets corresponding to the compounds were merged simultaneously, and the targets of HRG to treat NAFL through intestinal flora were obtained (Figure 4D). Module analysis and cytoHubba analysis were performed with this merged network. Module analysis shows module one (Figure 4E, score = 7.2) and module two (Figure 4F, score = 7.0), and cytoHubba analysis shows the top 10 targets (Figure 4G). Finally, 10 key potential therapeutic targets were obtained: CXCL10, CXCL8, ICAM1, IFNG, IL10, IL1B, IL2, IL4, IL6, TNF. The node size in Figures 4A–F is positively correlated with the degree value. Node colors in Figures 4B–F are positively correlated with degree values.

**TABLE 3:** Compound information in the top 20 nodes in the “herb-compound-target” network of HRG.

Number	Compound	TCMSP ID	Pubchem ID	Degree	OB(%)	DL	Herb source
HRG65	Quercetin	MOL000098	5280343	238	46.43	0.28	YC, SZ, NZZ, MHL, GQZ, CH, XJ, GC
HRG185	Kaempferol	MOL000422	5280863	158	41.88	0.24	DH, NZZ, CH, GC
HRG107	Luteolin	MOL000006	5280445	150	36.16	0.25	MHL, NZZ
HRG3	7-Methoxy-2-methyl isoflavone	MOL003896	354368	144	42.56	0.2	GC
HRG76	wogonin	MOL000173	5281703	142	30.68	0.23	CZ
HRG53	Glabridin	MOL004908	124052	138	53.25	0.47	GC
HRG81	7-Acetoxy-2-methylisoflavone	MOL004991	268208	136	38.92	0.26	GC
HRG189	licochalcone a	MOL000497	5318998	135	40.79	0.29	GC
HRG88	Glyasperins M	MOL005007	NA	134	72.67	0.59	GC
HRG74	4′-O-Methylglabridin	MOL004978	9927807	134	36.21	0.52	GC
HRG70	1-Methoxyphaseollidin	MOL004959	480873	134	69.98	0.64	GC
HRG86	Licoagrocarpin	MOL005003	15840593	133	58.81	0.58	GC
HRG239	Medicarpin	MOL002565	336327	133	49.22	0.34	GC
HRG179	isorhamnetin	MOL000354	5281654	133	49.6	0.31	YC, CH, GC

GO and KEGG enrichment analysis were performed for the intersection targets, and a total of 184 pathways (Supplementary Table S4) and 980 GO entries (Supplementary Table S5) were enriched. The KEGG enrichment results showed that there were 59 pathways related to Organic Systems, eight pathways related to Metabolism, 80 pathways related to Human Diseases, one pathway related to Genetic Information Processing, and 25 pathways related to Environmental Information Processing and 11 pathways related to Cellular Processes. The enrichment results showed that Cytokine-cytokine receptor interaction, Chagas disease, Influenza A and other pathways had better enrichment results. The GO enrichment results showed that the BP entries accounted for 97.04%, the MF entries accounted for 2.65%, and the CC entries accounted for 0.31%. BP mainly related to metabolic process of reactive oxygen species, response to molecules of bacterial origin, regulation of inflammatory response, etc. MF mainly related to Receptor ligand activity, signaling receptor activator activity, cytokine receptor binding, etc. CC mainly related to membrane raft, membrane microdomain and membrane region.

**FIGURE 5:**
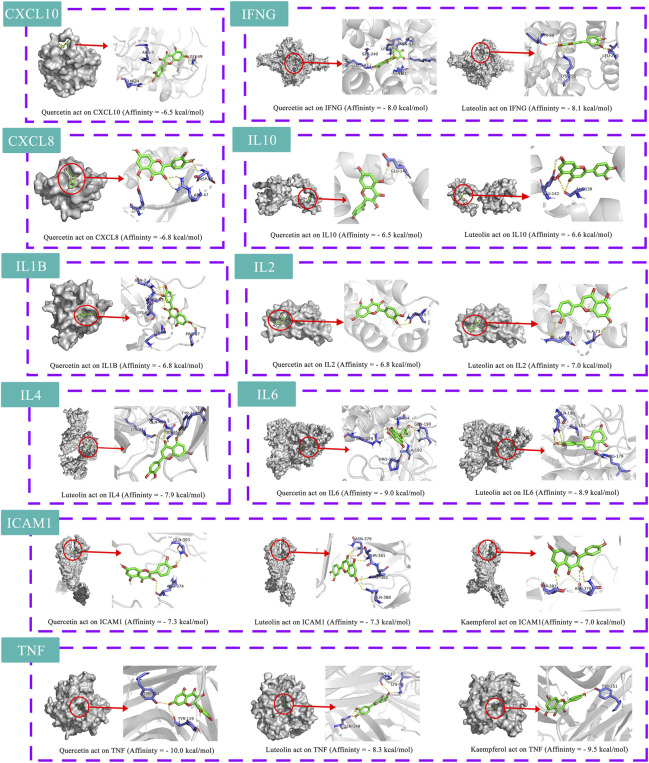
Molecular docking simulation.

The 10 key therapeutic targets, 14 key compounds, and related pathways were used to construct a network diagram of “herb-key compound-potential target-pathway” through Cytoscape (Figure 4H). The top three compounds were selected as potential compounds (quercetin, Luteolin, Kaempferol). The top three signaling pathways were selected as key pathways (Cytokine-cytokine receptor interaction, Chagas disease, IL-17 signaling pathway). Pathway Builder Tool 2.0 was used to draw the cartoon pathway mechanism of HRG in the treatment of NAFL through the intestinal flora (Figure 4I). The 10 key targets were molecularly docked with three potential compounds, and a total of 18 pairs of docking results were obtained (Table 4). The results were visualized using Pymol (Figure 5).

**TABLE 4:** Information of molecular docking.

Structure	PDB ID	Target	Compound ID	Compound name	Affinity (kcal/mol)
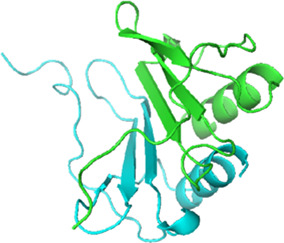	1O80	CXCL10	HRG65	Quercetin	−6.5
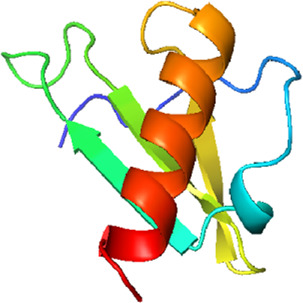	3IL8	CXCL8	HRG65	Quercetin	−6.8
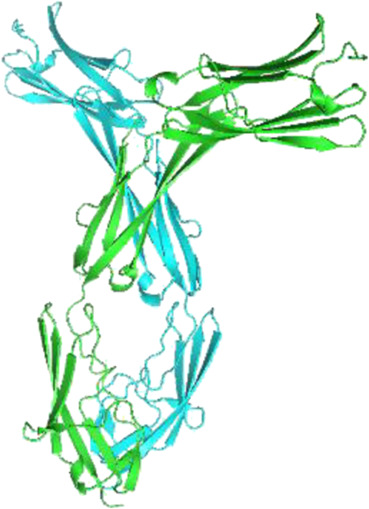	1P53	ICAM1	HRG107	Luteolin	−7.3
	1P53	ICAM1	HRG185	Kaempferol	−7
	1P53	ICAM1	HRG65	Quercetin	−7.3
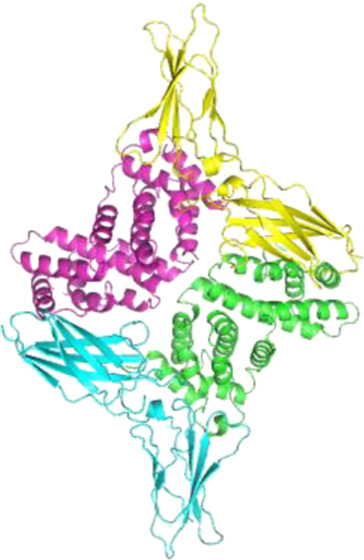	1FYH	IFNG	HRG107	Luteolin	−8.1
	1FYH	IFNG	HRG65	Quercetin	−8
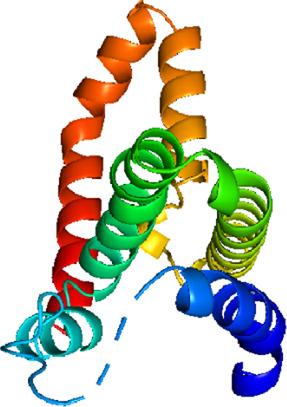	2H24	IL10	HRG107	Luteolin	−6.6
	2H24	IL10	HRG65	Quercetin	−6.5
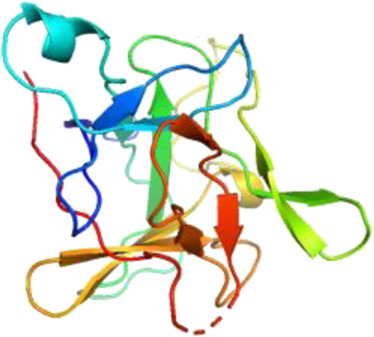	5R86	IL1B	HRG65	Quercetin	−6.8
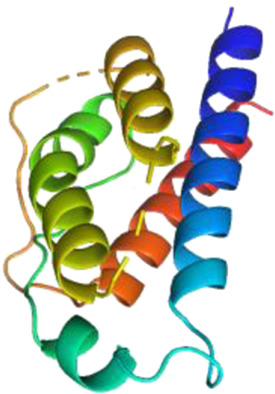	4NEM	IL2	HRG107	Luteolin	−7
	4NEM	IL2	HRG65	Quercetin	−6.8
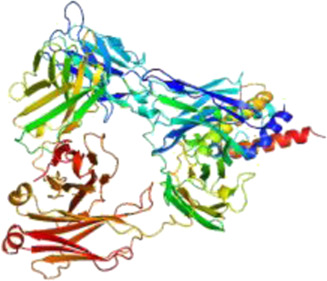	5FHX	IL4	HRG107	Luteolin	−7.9
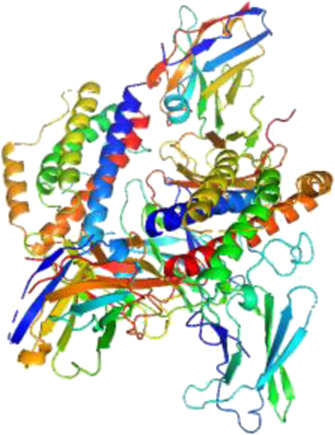	5FUC	IL6	HRG107	Luteolin	−8.9
	5FUC	IL6	HRG65	Quercetin	−9
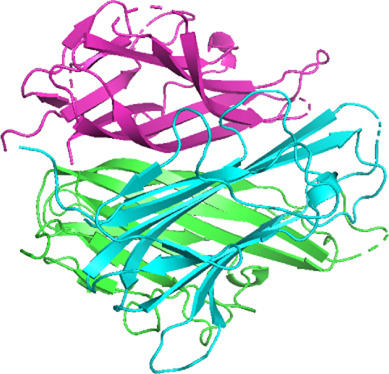	7KPA	TNF	HRG107	Luteolin	−8.3
	7KPA	TNF	HRG185	Kaempferol	−9.5
	7KPA	TNF	HRG65	Quercetin	−10

### Metagenomics Sequencing

The functional diversity of the environmental samples is analyzed from the perspective of gene function, and the species diversity contained in the environmental samples is analyzed from the perspective of species. The original sequence was filtered by fastp software to obtain high-quality sequencing data. Bowtie2 was used to align with the host genome sequence to remove host contamination. MEGAHIT software was used for macrogenome assembly, and contig sequences shorter than 300 bp were filtered. QUAST software was used to evaluate the assembly results. A total of 360,000,366,878 bp of clean reads were obtained through quality control, and the final number of effective reads was 914,019,667 bp. 15,830,378 contigs were obtained with a total length of 16,591,141,994 bp. MMseqs2 software was used to remove redundancy. The similarity threshold was set at 95% and the coverage threshold was set at 90%. The statistical results of the quality control of the sequencing data of each group can be found in Supplementary Table S6, and the statistical information of the assembly results can be found in Supplementary Table S7.

**FIGURE 6:**
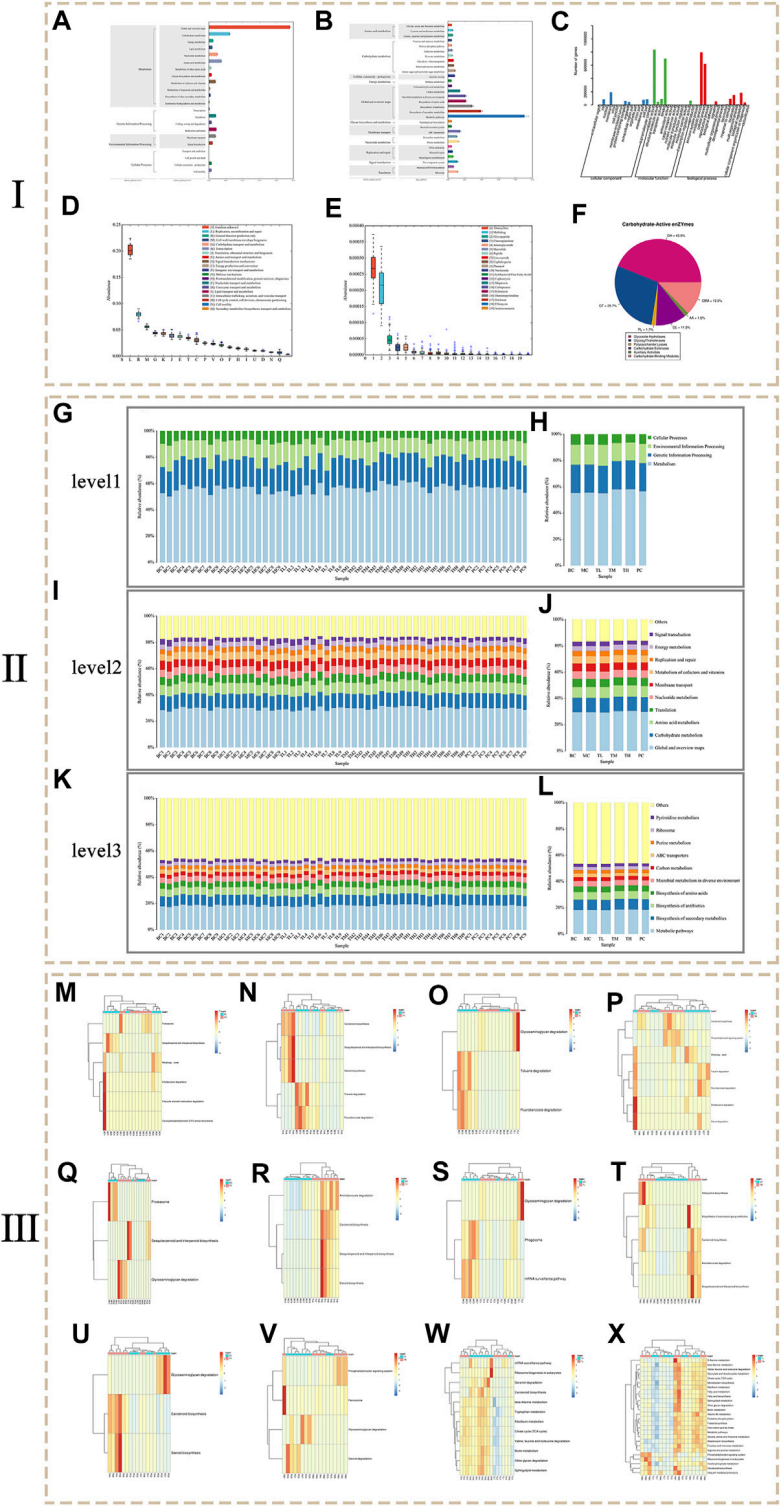
The results of metagenomics sequencing. (Ⅰ: Functional annotation analysis. Ⅱ: Histogram of KEGG pathway composition and abundance. Ⅲ: Heat map of metagenomeSeq differential pathway abundance).

Function annotation is based on the KEGG (Figures 6A,B), GO (Figure 6C), eggNOG (Figure 6D), CARD (Figure 6E), and CAZy (Figure 6F) databases. A total of 6055 KO (KEGG Ontology) and 2104 EC (enzyme) were annotated. Function was enriched in the pathways, resulting in four pathways at level 1, 22 pathways at level 2 and 167 pathways at level 3. The KEGG functional genes are closely related to metabolism related pathways, and the abundance of global and overview maps is highest for secondary pathways. Go annotated three categories, 42 secondary classifications, and 1,558 entries. EggNOG was annotated in four categories, 25 secondary classifications and 31039 eggNOG. The results showed that the number of corresponding functional genes of Replication, recommendation and repair and General function prediction only accounted for a relatively high proportion. Cell wall/membrane/envelope biogenesis and Carbohydrate transport and metabolism ranked second. The annotation results of the CARD database showed that the relative content of resistance genes corresponding to Tetracycline and Multidrug of antibiotic resistance is high, followed by Fluoroquinolone and Aminoglycoside. The annotation results of the CAZy database show that the top three carbohydrate enzymes are glycoside hydrolase (GH), glycosyltransferase (GT) and non-catalytic carbohydrate binding module (CBM).

Figures 6G–L shows the composition of the KEGG pathways at different levels of each group and sample. At level 1 of each group, the abundance of Metabolic was the highest and the abundance of Cellular processes was the lowest. At level 2 of each group, the abundance of Global and overview maps is the highest, followed by Carbohydrate metabolism and Amino acid metabolism. The abundances of Translation, Nucleoside metabolism, Membrane transport and Metabolism of cofactors and vitamins are similar. Among the levels 3 of each group, the abundance of metabolic pathways was the highest, followed by biosynthesis of secondary metals, and third was Biosynthesis of antibiotics. Biosynthesis of amino acids and Microbial metabolism in diverse environments have little difference in abundance. In contrast, Carbon metabolism, ABC transporters, Purine metabolism, Ribosome and Pyrimidine metabolism have little difference in abundance. The results showed that compared with the blank group and the model group, the abundance of Metabolism at level 1, Global and overview maps at level 2 and Metabolic pathways at level 3 increases to some extent in the HRG group. It is speculated that HRG can improve NAFL by increasing metabolic pathways associated with metabolic regulation.

MetagenomeSeq was used to test the difference in abundance of KEGG at level 3 (*p* < 0.05), and the analyzed difference was plotted according to abundance, as shown in Figures 6M–X. The results showed that the abundance of Proteasome in the MC group was significantly higher than that in the BC group, and the Sesquiterpenoid and triterpenoid biosynthesis in the MC group was significantly lower than that in the BC group. Compared with the MC group, the PC group changed this trend. Compared with the BC group, the abundance of Fluorobenzoate degradation and Toluene degradation was higher in the HRG group. Compared with the MC group, the metabolic pathways with higher abundance in the HRG group include Aminobenzoate degradation, Carotenoid biosynthesis, Sesquiterpenoid and triterpenoid biosynthesis, Steroid biosynthesis, Glycosaminoglycan degradation, Phagosome, mRNA surveillance pathway, Tetracycline biosynthesis, Biosynthesis of vancomycin group antibiotics, Carotenoid biosynthesis, Aminobenzoate degradation, Sesquiterpenoid and triterpenoid biosynthesis. Therefore, HRG may improve NAFL through Metabolism and Cellular processes in which metabolism related pathways play an important role. Aminobenzoate degradation, Steroid biosynthesis, and Sesquiterpenoid and triterpenoid biosynthesis are important metabolic pathways.

## Discussion

Nonalcoholic Fatty Liver (NAFL), a common chronic disease in the clinic, can develop into nonalcoholic steatohepatitis, which can progress to cirrhosis or even liver cancer and is also related with the occurrence and development of other chronic diseases (Wang and Malhi, 2018; Aron-Wisnewsky et al., 2020). When liver tissue is diseased or liver cells are damaged, the activity of related liver enzymes and serum concentrations, such as AKP, ALT and AST, is increased (Zhou et al., 2017; Eshraghian et al., 2020; Choudhary et al., 2021; Nielsen et al., 2021). NAFL itself is also a metabolic disease that may be manifested by abnormal lipid metabolism and a significant or insignificant increase in blood lipids, such as HDLC, LDL-C, TC and TG (Jimenez-Rivera et al., 2017; Chen et al., 2020; Liu et al., 2020; Mohammadi et al., 2020). In our study, serum HDL-C in model mice increased and decreased after administration, which was similar to the results of Sheng et al. (Sheng et al., 2019). Changes in serum levels may be related to the degree of NAFLD, species and individual differences. A number of studies have shown that adjuvant therapy regulated by intestinal flora can effectively improve liver function, blood lipids, BMI and other levels of NAFL patients, suggesting that intestinal flora plays an important role in NAFL treatment (Tsai et al., 2020; Burz et al., 2021; Fernandez-Botran et al., 2021; Pan et al., 2021; Yang et al., 2021). The results of this study showed that Huazhi Rougan granules (HRG) could improve liver enzymes and lipid indexes of NAFL mice. The improvement effect of HDL-C, TG and AKP was better than that of the positive control group, and the therapeutic effect was related to the concentration. In addition, HRG can improve the accumulation of liver lipids in NAFL model mice, and the improvement of liver lesions may be dose-dependent.

The results of microbial diversity analysis showed that HRG could increase microbial diversity, increase species richness, and affect the microbial structure of NAFL mice. At the phylum level, HRG increased *Bacteroides* and Verrucomicrobia and decreased Firmicutes and Proteobacteria in NAFL mice. At the genus level, Akkermansia increased, and *Lactobacillus* and Desulfovibrio reduced. RDA analysis showed that species distribution was significantly correlated with AKP, HDL-C and TG. Bacteroidetes were negatively correlated with AKP, AST, TG and TC, while Firmicutes were negatively correlated with ALT, LDL-C and TC. Other studies have also found that the intestinal flora composition affects NAFL (Rau et al., 2018; Demir et al., 2020). Studies have shown that the Firmicutes/Bacteroidetes (F/B) ratio is related not only to intestinal homeostasis but also related to NAFL (Porras et al., 2017; Lee et al., 2020; Stojanov et al., 2020; Jasirwan et al., 2021). The results of the animal experiment by Bao et al. were similar to ours, and the reduction of F/B ratio could improve NAFL-related indicators (Bao et al., 2020). Huang et al. ‘s experiment also showed that the reduction of F/B ratio could alleviate NAFL in mice (Huang et al., 2019). Metagenomic sequencing analysis is mainly concerned with functional information. The results showed that metabolism-related functions, such as metabolism-related pathways, including metabolic pathways, secondary metabolites, antibiotics and amino acid biosynthesis, were increased in the treatment group compared with the model group. The results showed that metabolism-related functions increased in the treatment group compared with the model group, and there were significant differences in metabolism-related pathways. Analysis of the differences in metabolic pathways showed that metabolism-related pathways differed significantly between groups, indicating that the metabolic activities of bacteria were vigorous. The application of HRG increased Aminobenzoate degradation, Steroid biosynthesis, Sesquiterpenoid and triterpenoid biosynthesis. Many sesquiterpenoids have been isolated from plants, fungi, marine organisms, and *Streptomyces* species (Bertea et al., 2006; Nagegowda et al., 2008; Field et al., 2011). Furthermore, sesquiterpene pyridine alkaloids from Tripterygium were predicted to target several proteins and signaling pathways and may play an important role in curing Alzheimer’s disease, Chagas disease, and NAFL (Long et al., 2021). The aminobenzoate degradation pathway could promote tryptophan metabolism and benzoate degradation (Naidu and Ragsdale, 2001; McLeish et al., 2003; Toraya et al., 2004). The animal studies by Zhang et al. showed that the main changes in fecal metabolites of high-fat fed mice included metabolites related to tryptophan metabolism (Zhang et al., 2021). Steroid biosynthesis is associated with lipid metabolism and may increase primary bile acid biosynthesis (Lange and Ghassemian, 2003; Morikawa et al., 2006). Studies have shown that key pathways of lipid metabolisms, such as steroid biosynthesis, fatty acid synthesis, and oxidation, are involved in the progression of non-alcoholic fatty liver disease (Huang et al., 2020). A number of studies have also shown that primary bile acid biosynthesis is closely related to the occurrence and development of non-alcoholic fatty liver disease, and inhibition of this metabolic pathway can improve lipid accumulation in NAFL (Zhong et al., 2018; Na et al., 2019; Zhang et al., 2020). Therefore, NAFL can be alleviated by decreasing the F/B ratio and increasing the corresponding metabolic pathways. These results suggest that HRG may regulate intestinal flora and improve lipid metabolism in NAFL mice through the above channels.

Three potential compounds (Quercetin, Luteolin, Kaempferol), 10 core targets (CXCL10, CXCL8, ICAM1, IFNG, IL10, IL1B, IL2, IL4, IL6, TNF) and three key pathways (Cytokine−cytokine receptor interaction, Chagas disease, IL-17 signaling pathway) were identified based on the network pharmacology results. The 10 identified core targets are mainly related to inflammation, and these cytokines play an important role in the inflammatory response and immune regulation (Ouyang and O'Garra, 2019; Torretta et al., 2020; Jurdziński et al., 2020; Bui et al., 2020; Zhong et al., 2020). A number of studies have shown that the occurrence and progression of NAFL are closely related to the increased expression level of inflammatory factors (Chakraborty et al., 2019; Her et al., 2020; Moreno-Fernandez et al., 2021). The rat experiments by Ogunlana et al. showed that inhibition of oxidative stress, increase in antioxidant enzyme levels, and decrease in pro-inflammatory markers (IL-2, IL-6, TNF-α) could reverse NAFL-induced histological changes in the liver in rats (Ogunlana et al., 2020). Other studies have reported that these cytokines are associated with intestinal microflora disorder, intestinal immunity and intestinal inflammation (Sun et al., 2020). For example, Hui et al., found that alterations in the bacterial flora of neonatal necrotizing enterocolitis patients resulted in overt intestinal inflammation and increased expression of IL-1, IL-2, IL-4, IL-6, IL-8, IL-10, TNF-α, IFN-γ and IL-17 in the samples (Hui et al., 2017). Other experiments have also proved that inflammatory factors have an important relationship with intestinal flora disorder in NAFL mice, and improving intestinal flora disorder may intervene in the occurrence and progression of NAFL (Zhang et al., 2018; Li et al., 2020; Kang et al., 2021; Li et al., 2021; Shi et al., 2021). Studies have shown that Chagas disease is related to the changes and dysfunction of intestinal microbiota, which may be an important mechanism for the occurrence of intestinal flora disorder (de Souza-Basqueira et al., 2020; Duarte-Silva et al., 2020; Schaub, 2021). The study by Wang et al. suggests that the differentiation of cytokines related to the IL-17 signaling pathway is related to the colonization of intestinal flora and may be involved in intestinal immune homeostasis (Wang et al., 2019). The animal experiments of Xin et al. and the mass spectrometry analysis Du et al. showed that both the enterohepatic circulation and the intestinal flora are involved in the absorption process of quercetin and kaempferol and that isorhamnetin 3-O-glucoside could be successively decomposed into quercetin and kaempferol under bacterial action (Du et al., 2014; Du et al., 2017; Xin et al., 2019). A number of animal experiments have shown that quercetin can improve hepatic steatosis and prevent hepatic lipid accumulation, thereby treating NAFL (Esrefoglu et al., 2017; Donaldson et al., 2019). Therefore, we believe that HRG may improve intestinal inflammation, intestinal immunity, and lipid synthesis through relevant pathways, thereby improving NAFL by regulating intestinal flora. However, our study has some limitations, and more studies are needed for further verification.

## Conclusion

In conclusion, HRG may alter microbial diversity, structure, and function to improve NAFL induced by high-fat diets. It is also possible to improve NAFL-related lipid accumulation and liver lesions by regulating intestinal related metabolic pathways, inflammatory responses, and immune responses. These results strongly suggest that HRG may alleviate NAFL by preventing intestinal flora disorder. Moreover, HRG can treat NAFL with multiple components, multiple metabolic pathways, and multiple targets through intestinal flora. This study provides a new idea for the treatment of NAFL, proves the relationship between intestinal flora and NAFL, and suggests that HRG has a good therapeutic effect.

## Data Availability

The datasets presented in this study can be found in online repositories. The names of the repository/repositories and accession number(s) can be found below: https://www.ncbi.nlm.nih.gov/; 16S rRNA sequencing data is available *via* BioProject PRJNA807481, metagenomic sequencing data is available *via* BioProject PRJNA807437.
