# Corrigendum: Revealing the mechanism of Huazhi Rougan granule in the treatment of nonalcoholic fatty liver through intestinal flora based on 16S rRNA, metagenomic sequencing and network pharmacology

**DOI:** 10.3389/fphar.2023.1243304

**Published:** 2023-07-26

**Authors:** Yingying Liu, Yingying Tan, Jiaqi Huang, Chao Wu, Xiaotian Fan, Antony Stalin, Shan Lu, Haojia Wang, Jingyuan Zhang, Fanqin Zhang, Zhishan Wu, Bing Li, Zhihong Huang, Meilin Chen, Guoliang Cheng, Yanfang Mou, Jiarui Wu

**Affiliations:** ^1^ Department of Clinical Chinese Pharmacy, School of Chinese Materia Medica, Beijing University of Chinese Medicine, Beijing, China; ^2^ Institute of Fundamental and Frontier Sciences, University of Electronic Science and Technology of China, Chengdu, China; ^3^ State Key Laboratory of Generic Manufacture Technology of Chinese Traditional Medicine, Linyi, China

**Keywords:** nonalcoholic simple fatty liver, high-fat diet, intestinal flora disorder, 16S sequencing, network pharmacology

## Abstract

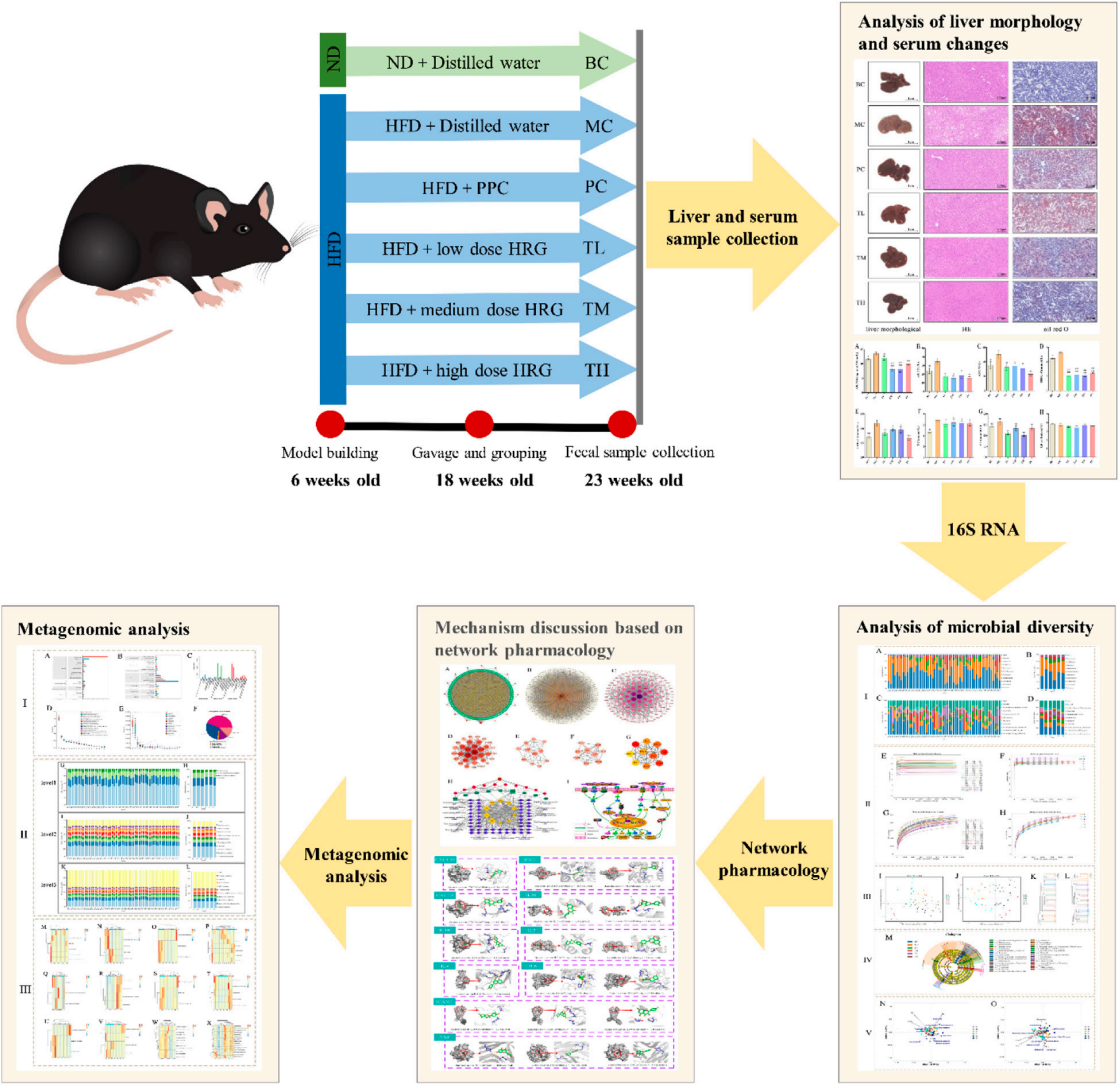

In the published article, there was an error in the **Graphical Abstract** as published. The number of weeks in the picture are incorrect. The **Graphical Abstract** has been corrected.

Additionally, a correction has been made to the section **Abstract**, subsection *Methods*, 2nd sentence. This sentence previously stated:

“In this study, C57BL/6J mice were fed a high-fat diet for 8 weeks, and the high-fat diet plus HRG or polyene phosphatidylcholine capsules were each administered by gavage for 4 weeks.”

The corrected sentence appears below:

“In this study, C57BL/6J mice were fed a high-fat diet for 10 weeks, and the high-fat diet plus HRG or polyene phosphatidylcholine capsules were each administered by gavage for 5 weeks.”

Lastly, a correction has been made to section **Materials and Methods**, subsection *Animal Grouping and Model Establishment*, 2nd and 3rd sentences. These sentences previously stated:

“After 12 consecutive weeks, relevant indicators were evaluated. Mice that passed the evaluation could be considered successful modeling, gavage at week 13, and sampled at week 17.”

The corrected sentences appear below:

“After 10 consecutive weeks, relevant indicators were evaluated. Mice that passed the evaluation could be considered successful modeling, gavage at week 12, and sampled at week 17.”

The authors apologize for these errors and state that this does not change the scientific conclusions of the article in any way. The original article has been updated.

