# Supplementary material for: Revealing the Mechanism of Huazhi Rougan Granule in the Treatment of Nonalcoholic Fatty Liver Through Intestinal Flora Based on 16S rRNA, Metagenomic Sequencing and Network Pharmacology
Source: Front Pharmacol. 2022 Apr 26;13:875700. doi: 10.3389/fphar.2022.875700 (PMC9086680; doi:10.3389/fphar.2022.875700)
Supplement: Supplementary file 1 [file Table1.DOCX]

Additional file 1 Quality evaluation of sequencing data. (‾x±se, n=9)

| Index/Group | BC | MC | TL | TM | TH | PC |
| --- | --- | --- | --- | --- | --- | --- |
| Raw Reads | 69112.78±4659.03 | 76570.78±3248.10 | 80114.00±72.59 | 79935.33±57.14 | 79995.78±66.01 | 79998.56±47.37 |
| Clean Reads | 68895.67±4649.07 | 76329.89±3239.22 | 79858.11±74.52 | 79693.11±62.05 | 79756.44±70.07 | 79767.00±46.48 |
| Effective Reads | 68160.22±4600.77 | 75185.22±3148.08 | 78405.00±244.26 | 77619.00±549.81 | 78608.67±168.43 | 77777.00±810.77 |
| AvgLen(bp) | 418.00±1.04 | 419.22±0.91 | 418.78±1.01 | 416.89±0.92 | 418.33±0.91 | 417.56±1.00 |
| GC(%) | 54.41±0.28 | 54.62±0.17 | 55.25±0.23 | 54.42±0.12 | 55.31±0.19 | 55.20±0.29 |
| Q20(%) | 99.09±0.01 | 99.10±0.01 | 99.10±0.01 | 99.12±0.01 | 99.13±0.01 | 99.13±0.01 |
| Q30(%) | 96.23±0.03 | 96.26±0.04 | 96.23±0.05 | 96.35±0.04 | 96.36±0.03 | 96.36±0.04 |
| Effective(%) | 98.61±0.13 | 98.22±0.28 | 97.87±0.29 | 97.10±0.70 | 98.27±0.19 | 97.22±1.04 |
