# Supplementary material for: Revealing the Mechanism of Huazhi Rougan Granule in the Treatment of Nonalcoholic Fatty Liver Through Intestinal Flora Based on 16S rRNA, Metagenomic Sequencing and Network Pharmacology
Source: Front Pharmacol. 2022 Apr 26;13:875700. doi: 10.3389/fphar.2022.875700 (PMC9086680; doi:10.3389/fphar.2022.875700)
Supplement: Supplementary file 2 [file Table7.DOCX]

Additional file 7 Statistical results of macrogenome assembly

| Group/Index | Contig Num. | Total Len.(bp) | Largest Len.(bp) | N50(bp) | GC(%) | Mapped(%) |
| --- | --- | --- | --- | --- | --- | --- |
| BC | 277258.89±7819.62 | 285639719.78±7743420.44 | 498015.56±44490.05 | 1558.44±48.65 | 47.45±0.18 | 86.48±1.26 |
| MC | 265437.22±9107.80 | 284908029.11±6701428.39 | 377244.67±48147.18 | 1785.56±133.44 | 48.51±0.17 | 85.79±1.31 |
| TL | 285404.78±18470.95 | 318048738.56±18171614.66 | 334740.22±21328.80 | 1931.44±118.87 | 48.13±0.26 | 88.87±0.86 |
| TM | 269161.44±22959.77 | 294426361.44±17726282.07 | 308876.22±22582.69 | 1998.33±208.30 | 47.99±0.29 | 86.78±1.59 |
| TH | 325594.56±25209.08 | 332164196.22±21792137.85 | 282556.33±26320.28 | 1606.78±70.82 | 48.30±0.19 | 90.50±0.59 |
| PC | 336074±12035.18 | 328273176.44±13747825.26 | 278404.22±26900.67 | 1456.44±64.43 | 48.63±0.26 | 89.83±0.70 |
