# Supplementary material for: Revealing the Mechanism of Huazhi Rougan Granule in the Treatment of Nonalcoholic Fatty Liver Through Intestinal Flora Based on 16S rRNA, Metagenomic Sequencing and Network Pharmacology
Source: Front Pharmacol. 2022 Apr 26;13:875700. doi: 10.3389/fphar.2022.875700 (PMC9086680; doi:10.3389/fphar.2022.875700)
Supplement: Supplementary file 3 [file Table2.DOCX]

Additional file 2 Metastats analysis difference data statistics of phylum level

| Group | phylum | Q value |
| --- | --- | --- |
| BC_MC | Acidobacteria | 0.011322 |
|  | Actinobacteria | 0.011322 |
|  | Planctomycetes | 0.011322 |
|  | Firmicutes | 0.033966 |
|  | Bacteroidetes | 0.033966 |
|  | Proteobacteria | 0.045288 |
| BC_PC | Actinobacteria | 1.70E-02 |
|  | Planctomycetes | 1.70E-02 |
|  | Tenericutes | 3.40E-02 |
|  | Bacteroidetes | 4.25E-02 |
| BC_TL | Actinobacteria | 0.033966 |
|  | Acidobacteria | 0.033966 |
| BC_TH | Planctomycetes | 1.70E-02 |
|  | Actinobacteria | 1.70E-02 |
| BC_TM | Planctomycetes | 1.70E-02 |
|  | Cyanobacteria | 1.70E-02 |
| MC_TH | Gemmatimonadetes | 5.66E-03 |
|  | Nitrospirae | 5.66E-03 |
|  | Verrucomicrobia | 5.66E-03 |
|  | Firmicutes | 8.49E-03 |
|  | Bacteroidetes | 3.40E-02 |
|  | Epsilonbacteraeota | 4.81E-02 |
| MC_TM | Gemmatimonadetes | 8.49E-03 |
|  | Nitrospirae | 8.49E-03 |
|  | Cyanobacteria | 1.27E-02 |
|  | Verrucomicrobia | 1.27E-02 |
|  | Rokubacteria | 1.36E-02 |
|  | Actinobacteria | 2.26E-02 |
| MC_PC | Gemmatimonadetes | 8.49E-03 |
|  | Nitrospirae | 8.49E-03 |
|  | Verrucomicrobia | 2.26E-02 |
|  | Tenericutes | 4.76E-02 |
|  | Rokubacteria | 4.76E-02 |
| MC_TL | Planctomycetes | 0.016983 |
| PC_TH | Tenericutes | 0.022644 |
|  | Bacteroidetes | 0.022644 |
|  | Epsilonbacteraeota | 0.022644 |
|  | Firmicutes | 0.025475 |
| PC_TM | Actinobacteria | 3.40E-02 |
| TL_TH | Planctomycetes | 1.70E-02 |
| TL_TM | Planctomycetes | 1.70E-02 |
|  | Gemmatimonadetes | 2.26E-02 |
|  | Cyanobacteria | 2.26E-02 |
|  | Actinobacteria | 2.55E-02 |
|  | Rokubacteria | 4.08E-02 |
| PC_TL | Planctomycetes | 0.016983 |
|  | Patescibacteria | 0.042458 |
