# Supplementary material for: Revealing the Mechanism of Huazhi Rougan Granule in the Treatment of Nonalcoholic Fatty Liver Through Intestinal Flora Based on 16S rRNA, Metagenomic Sequencing and Network Pharmacology
Source: Front Pharmacol. 2022 Apr 26;13:875700. doi: 10.3389/fphar.2022.875700 (PMC9086680; doi:10.3389/fphar.2022.875700)
Supplement: Supplementary file 5 [file Table3.DOCX]

Additional file 3 Metastats analysis difference data statistics of genus level

| Group | genus | Q value | Group | genus | Q value |
| --- | --- | --- | --- | --- | --- |
| BC_MC | uncultured_bacterium_o_Clostridiales | 0.04 | MC_TM | Adlercreutzia | 0.00747 |
| BC_MC | uncultured_bacterium_f_Mitochondria | 0.0208 | MC_TM | Allobaculum | 0.00747 |
| BC_MC | uncultured_bacterium_f_Erysipelotrichaceae | 0.0156 | MC_TM | Bacteroides | 0.00747 |
| BC_MC | uncultured_bacterium_f_Desulfovibrionaceae | 0.0156 | MC_TM | Coprobacillus | 0.00747 |
| BC_MC | uncultured_bacterium_f_Blastocatellaceae | 0.0346 | MC_TM | Dongia | 0.00747 |
| BC_MC | uncultured_bacterium_f_Atopobiaceae | 0.0156 | MC_TM | Dubosiella | 0.00747 |
| BC_MC | UBA1819 | 0.0156 | MC_TM | Enterorhabdus | 0.00747 |
| BC_MC | Tyzzerella_3 | 0.0156 | MC_TM | Gardnerella | 0.00747 |
| BC_MC | Stenotrophobacter | 0.0208 | MC_TM | Listeria | 0.00747 |
| BC_MC | Sphingomonas | 0.0346 | MC_TM | MND1 | 0.00747 |
| BC_MC | Singulisphaera | 0.0208 | MC_TM | Nitrospira | 0.00747 |
| BC_MC | Ruminococcaceae_UCG-013 | 0.0156 | MC_TM | Parabacteroides | 0.00747 |
| BC_MC | Ruminococcaceae_UCG-010 | 0.0346 | MC_TM | RB41 | 0.00747 |
| BC_MC | Rikenellaceae_RC9_gut_group | 0.0156 | MC_TM | Sphingomonas | 0.00747 |
| BC_MC | Phenylobacterium | 0.0208 | MC_TM | Steroidobacter | 0.00747 |
| BC_MC | Odoribacter | 0.0208 | MC_TM | UBA1819 | 0.00747 |
| BC_MC | Millionella | 0.0451 | MC_TM | uncultured_bacterium_c_Bacteroidia | 0.00747 |
| BC_MC | Methylobacterium | 0.0346 | MC_TM | uncultured_bacterium_c_Deltaproteobacteria | 0.00747 |
| BC_MC | Lactobacillus | 0.0156 | MC_TM | uncultured_bacterium_c_Subgroup_6 | 0.00747 |
| BC_MC | Gardnerella | 0.0346 | MC_TM | uncultured_bacterium_f_Blastocatellaceae | 0.00747 |
| BC_MC | Enterorhabdus | 0.0346 | MC_TM | uncultured_bacterium_f_Clostridium_sp._K4410.MGS-306 | 0.00747 |
| BC_MC | Dongia | 0.0156 | MC_TM | uncultured_bacterium_f_Gemmatimonadaceae | 0.00747 |
| BC_MC | Bilophila | 0.0156 | MC_TM | uncultured_bacterium_o_Azospirillales | 0.00747 |
| BC_MC | Bifidobacterium | 0.0156 | MC_TM | uncultured_bacterium_o_Gastranaerophilales | 0.00747 |
| BC_MC | Bacteroides | 0.0156 | MC_TM | uncultured_bacterium_o_Rokubacteriales | 0.00747 |
| BC_MC | Anaeroplasma | 0.0295 | MC_TM | Akkermansia | 0.0133 |
| BC_MC | Acidisphaera | 0.0208 | MC_TM | Escherichia-Shigella | 0.0133 |
| BC_MC | Acidipila | 0.0346 | MC_TM | uncultured_bacterium_o_Rhodospirillales | 0.0133 |
| BC_MC | [Eubacterium]_brachy_group | 0.0346 | MC_TM | GCA-900066575 | 0.0181 |
| BC_PC | uncultured_bacterium_f_Rhodobacteraceae | 0.0156 | MC_TM | Turicibacter | 0.0181 |
| BC_PC | uncultured_bacterium_f_Christensenellaceae | 0.0156 | MC_TM | [Ruminococcus]_torques_group | 0.0181 |
| BC_PC | uncultured_bacterium_f_Atopobiaceae | 0.0156 | MC_TM | Blautia | 0.0214 |
| BC_PC | uncultured_bacterium_f_Acetobacteraceae | 0.0156 | MC_TM | Chroococcidiopsis_PCC_7203 | 0.0214 |
| BC_PC | Tyzzerella_3 | 0.0156 | MC_TM | DNF00809 | 0.0214 |
| BC_PC | Succinivibrio | 0.044 | MC_TM | uncultured_bacterium_f_Sphingomonadaceae | 0.0214 |
| BC_PC | Staphylococcus | 0.0267 | MC_TM | Gordonibacter | 0.0259 |
| BC_PC | Singulisphaera | 0.0156 | MC_TM | Ruminococcaceae_UCG-010 | 0.0295 |
| BC_PC | Paeniclostridium | 0.044 | MC_TM | Ruminococcaceae_UCG-013 | 0.0295 |
| BC_PC | Methylobacterium | 0.0156 | MC_TM | Tyzzerella_3 | 0.0297 |
| BC_PC | Dubosiella | 0.0267 | MC_TM | Ruminococcus_1 | 0.0319 |
| BC_PC | Burkholderia-Caballeronia-Paraburkholderia | 0.0374 | MC_TM | Stenotrophobacter | 0.0319 |
| BC_PC | Bifidobacterium | 0.0156 | MC_TM | Rikenellaceae_RC9_gut_group | 0.04 |
| BC_PC | Alloprevotella | 0.0156 | MC_TM | Ruminococcaceae_UCG-005 | 0.0434 |
| BC_PC | Adlercreutzia | 0.0156 | MC_TM | Acetatifactor | 0.0467 |
| BC_PC | Acidisphaera | 0.0156 | MC_TH | RB41 | 0.007185 |
| BC_PC | Acidipila | 0.0156 | MC_TH | Nitrospira | 0.007185 |
| BC_TH | Acidisphaera | 0.0234 | MC_TH | Roseiarcus | 0.007185 |
| BC_TH | Adlercreutzia | 0.0234 | MC_TH | MND1 | 0.007185 |
| BC_TH | Odoribacter | 0.0234 | MC_TH | Listeria | 0.007185 |
| BC_TH | Tyzzerella_3 | 0.0234 | MC_TH | Lactobacillus | 0.007185 |
| BC_TH | uncultured_bacterium_c_Bacteroidia | 0.0234 | MC_TH | Ruminococcaceae_UCG-010 | 0.007185 |
| BC_TH | uncultured_bacterium_f_Acetobacteraceae | 0.0234 | MC_TH | Ruminococcaceae_UCG-013 | 0.007185 |
| BC_TH | uncultured_bacterium_o_Gammaproteobacteria_Incertae_Sedis | 0.0234 | MC_TH | Sphingomonas | 0.007185 |
| BC_TH | uncultured_bacterium_o_Rhodospirillales | 0.0234 | MC_TH | Turicibacter | 0.007185 |
| BC_TH | Acidipila | 0.0249 | MC_TH | UBA1819 | 0.007185 |
| BC_TH | Granulicella | 0.0249 | MC_TH | uncultured_bacterium_c_Bacteroidia | 0.007185 |
| BC_TH | Occallatibacter | 0.0249 | MC_TH | Rikenellaceae_RC9_gut_group | 0.007185 |
| BC_TH | Singulisphaera | 0.0249 | MC_TH | uncultured_bacterium_c_Deltaproteobacteria | 0.007185 |
| BC_TH | uncultured_bacterium_f_Atopobiaceae | 0.0249 | MC_TH | Dongia | 0.007185 |
| BC_TH | uncultured_bacterium_f_Desulfovibrionaceae | 0.0249 | MC_TH | uncultured_bacterium_f_Acetobacteraceae | 0.007185 |
| BC_TH | uncultured_bacterium_f_Xanthobacteraceae | 0.0249 | MC_TH | Acidothermus | 0.007185 |
| BC_TH | Bifidobacterium | 0.0295 | MC_TH | Adlercreutzia | 0.007185 |
| BC_TH | Methylobacterium | 0.0295 | MC_TH | Akkermansia | 0.007185 |
| BC_TH | Reyranella | 0.0295 | MC_TH | uncultured_bacterium_o_Gammaproteobacteria_Incertae_Sedis | 0.007185 |
| BC_TH | uncultured_bacterium_o_Clostridiales | 0.0295 | MC_TH | uncultured_bacterium_o_Azospirillales | 0.007185 |
| BC_TH | Clostridium_sensu_stricto_1 | 0.0356 | MC_TH | uncultured_bacterium_c_Subgroup_6 | 0.007185 |
| BC_TH | Roseiarcus | 0.0356 | MC_TH | uncultured_bacterium_o_Rhodospirillales | 0.007185 |
| BC_TH | Romboutsia | 0.0389 | MC_TH | uncultured_bacterium_f_Gemmatimonadaceae | 0.007185 |
| BC_TH | uncultured_bacterium_f_Rhodobacteraceae | 0.0389 | MC_TH | uncultured_bacterium_f_Erysipelotrichaceae | 0.007185 |
| BC_TH | uncultured_bacterium_o_Subgroup_2 | 0.0389 | MC_TH | Anaerotruncus | 0.007185 |
| BC_TH | Acidothermus | 0.04 | MC_TH | Steroidobacter | 0.011676 |
| BC_TH | Burkholderia-Caballeronia-Paraburkholderia | 0.04 | MC_TH | Candidatus_Solibacter | 0.011676 |
| BC_TH | Candidatus_Solibacter | 0.04 | MC_TH | Ruminiclostridium_6 | 0.011676 |
| BC_TH | Lachnospiraceae_UCG-001 | 0.04 | MC_TH | uncultured_bacterium_f_Clostridium_sp._K4410.MGS-306 | 0.011676 |
| BC_TL | Adlercreutzia | 0.026688 | MC_TH | uncultured_bacterium_f_Blastocatellaceae | 0.011676 |
| BC_TL | Lachnospiraceae_XPB1014_group | 0.026688 | MC_TH | Ruminococcaceae_UCG-004 | 0.011676 |
| BC_TL | Bifidobacterium | 0.026688 | MC_TH | uncultured_bacterium_o_Subgroup_2 | 0.016484 |
| BC_TL | Rikenellaceae_RC9_gut_group | 0.026688 | MC_TH | Occallatibacter | 0.016484 |
| BC_TL | Gordonibacter | 0.026688 | MC_TH | Desulfovibrio | 0.020196 |
| BC_TL | Romboutsia | 0.026688 | MC_TH | uncultured_bacterium_f_Clostridiales_vadinBB60_group | 0.020196 |
| BC_TL | uncultured_bacterium_f_Desulfovibrionaceae | 0.026688 | MC_TH | Anaeroplasma | 0.020196 |
| BC_TL | Phenylobacterium | 0.033966 | MC_TH | Lachnospiraceae_NK4A136_group | 0.02395 |
| BC_TL | Millionella | 0.033966 | MC_TH | uncultured_bacterium_f_Peptococcaceae | 0.02395 |
| BC_TL | Alistipes | 0.033966 | MC_TH | Bacteroides | 0.028022 |
| BC_TL | Lachnospiraceae_UCG-001 | 0.033966 | MC_TH | Granulicella | 0.031895 |
| BC_TL | Anaeroplasma | 0.046703 | MC_TH | uncultured_bacterium_f_Christensenellaceae | 0.035583 |
| BC_TM | Acidipila | 0.026688 | MC_TH | Negativibacillus | 0.0391 |
| BC_TM | Acidisphaera | 0.026688 | MC_TH | Helicobacter | 0.042458 |
| BC_TM | Lachnospiraceae_UCG-001 | 0.026688 | MC_TH | uncultured_bacterium_f_Muribaculaceae | 0.042811 |
| BC_TM | Adlercreutzia | 0.026688 | MC_TH | DNF00809 | 0.042811 |
| BC_TM | Singulisphaera | 0.026688 | MC_TH | Parasutterella | 0.042811 |
| BC_TM | Granulicella | 0.026688 | MC_TH | Dorea | 0.042811 |
| BC_TM | Gordonibacter | 0.026688 | MC_TH | Stenotrophobacter | 0.04575 |
| BC_TM | Bilophila | 0.041514 | MC_TL | Adlercreutzia | 0.0311 |
| BC_TM | Odoribacter | 0.041514 | MC_TL | Lachnospiraceae_XPB1014_group | 0.0311 |
| BC_TM | Lachnoclostridium | 0.046703 | MC_TL | Singulisphaera | 0.0311 |
| BC_TM | uncultured_bacterium_f_Desulfovibrionaceae | 0.046703 | MC_TL | Sphingomonas | 0.0311 |
| BC_TM | uncultured_bacterium_o_Clostridiales | 0.046703 | MC_TL | Tyzzerella_3 | 0.0311 |
| MC_PC | Acidisphaera | 0.0104 | MC_TL | UBA1819 | 0.0311 |
| MC_PC | Adlercreutzia | 0.0104 | TH_TL | Burkholderia-Caballeronia-Paraburkholderia | 0.0187 |
| MC_PC | Anaerotruncus | 0.0104 | TH_TL | Granulicella | 0.0187 |
| MC_PC | Dongia | 0.0104 | TH_TL | Listeria | 0.0187 |
| MC_PC | Gardnerella | 0.0104 | TH_TL | Parabacteroides | 0.0187 |
| MC_PC | Lachnospiraceae_UCG-006 | 0.0104 | TH_TL | RB41 | 0.0187 |
| MC_PC | Listeria | 0.0104 | TH_TL | Rikenellaceae_RC9_gut_group | 0.0187 |
| MC_PC | MND1 | 0.0104 | TH_TL | Ruminococcaceae_UCG-002 | 0.0187 |
| MC_PC | Nitrospira | 0.0104 | TH_TL | Singulisphaera | 0.0187 |
| MC_PC | RB41 | 0.0104 | TH_TL | uncultured_bacterium_c_Deltaproteobacteria | 0.0187 |
| MC_PC | Ruminiclostridium_5 | 0.0104 | TH_TL | uncultured_bacterium_f_Acetobacteraceae | 0.0187 |
| MC_PC | Steroidobacter | 0.0104 | TH_TL | Acidipila | 0.034 |
| MC_PC | UBA1819 | 0.0104 | TH_TL | Roseiarcus | 0.0374 |
| MC_PC | uncultured_bacterium_c_Deltaproteobacteria | 0.0104 | TH_TL | Ruminiclostridium_6 | 0.0374 |
| MC_PC | uncultured_bacterium_c_Subgroup_6 | 0.0104 | TH_TL | uncultured_bacterium_c_Subgroup_6 | 0.0374 |
| MC_PC | uncultured_bacterium_f_Acetobacteraceae | 0.0104 | TH_TL | uncultured_bacterium_f_Gemmatimonadaceae | 0.0374 |
| MC_PC | uncultured_bacterium_f_Christensenellaceae | 0.0104 | TH_TL | Lachnospiraceae_XPB1014_group | 0.0374 |
| MC_PC | uncultured_bacterium_o_Azospirillales | 0.0104 | TH_TL | Anaeroplasma | 0.0374 |
| MC_PC | Staphylococcus | 0.0187 | TH_TL | Millionella | 0.0374 |
| MC_PC | uncultured_bacterium_o_Mollicutes_RF39 | 0.0187 | TH_TL | uncultured_bacterium_o_Rhodospirillales | 0.0374 |
| MC_PC | uncultured_bacterium_f_Gemmatimonadaceae | 0.0267 | TH_TL | uncultured_bacterium_o_Subgroup_2 | 0.0374 |
| MC_PC | Intestinimonas | 0.034 | TH_TL | Acidisphaera | 0.0445 |
| MC_PC | Gordonibacter | 0.0406 | TH_TL | Alistipes | 0.0467 |
| PC_TM | Allobaculum | 0.0374 | TH_TL | Candidatus_Stoquefichus | 0.0467 |
| PC_TM | Alloprevotella | 0.0374 | TH_TL | uncultured_bacterium_f_Clostridiales_vadinBB60_group | 0.0467 |
| PC_TM | Dubosiella | 0.0374 | TL_TM | Bacteroides | 0.0144 |
| PC_TM | uncultured_bacterium_f_Acetobacteraceae | 0.0374 | TL_TM | Burkholderia-Caballeronia-Paraburkholderia | 0.0144 |
| PC_TM | uncultured_bacterium_f_Christensenellaceae | 0.0374 | TL_TM | Dubosiella | 0.0144 |
| PC_TM | Enterorhabdus | 0.0467 | TL_TM | Gardnerella | 0.0144 |
| PC_TM | Gemella | 0.0467 | TL_TM | Granulicella | 0.0144 |
| PC_TM | uncultured_bacterium_f_Clostridium_sp._K4410.MGS-306 | 0.0467 | TL_TM | Lachnoclostridium | 0.0144 |
| PC_TL | Acidipila | 0.016983 | TL_TM | Lachnospiraceae_XPB1014_group | 0.0144 |
| PC_TL | Acidisphaera | 0.016983 | TL_TM | Listeria | 0.0144 |
| PC_TL | Acinetobacter | 0.016983 | TL_TM | Ruminococcaceae_UCG-002 | 0.0144 |
| PC_TL | uncultured_bacterium_f_Acetobacteraceae | 0.016983 | TL_TM | Singulisphaera | 0.0144 |
| PC_TL | Burkholderia-Caballeronia-Paraburkholderia | 0.016983 | TL_TM | UBA1819 | 0.0144 |
| PC_TL | Gardnerella | 0.016983 | TL_TM | uncultured_bacterium_f_Clostridium_sp._K4410.MGS-306 | 0.0144 |
| PC_TL | Lachnospiraceae_XPB1014_group | 0.016983 | TL_TM | uncultured_bacterium_o_Gastranaerophilales | 0.0144 |
| PC_TL | Tyzzerella_3 | 0.016983 | TL_TM | Alistipes | 0.0197 |
| PC_TL | Listeria | 0.016983 | TL_TM | Bifidobacterium | 0.0197 |
| PC_TL | uncultured_bacterium_f_Christensenellaceae | 0.016983 | TL_TM | Erysipelatoclostridium | 0.0197 |
| PC_TL | Singulisphaera | 0.016983 | TL_TM | Faecalibaculum | 0.0197 |
| PC_TL | Candidatus_Saccharimonas | 0.031136 | TL_TM | Parabacteroides | 0.0197 |
| PC_TL | Gemella | 0.040031 | TL_TM | uncultured_bacterium_c_Deltaproteobacteria | 0.0197 |
| PC_TL | Pantoea | 0.040031 | TL_TM | RB41 | 0.028 |
| PC_TL | Stenotrophomonas | 0.049817 | TL_TM | Acidipila | 0.034 |
|  |  |  | TL_TM | uncultured_bacterium_f_Gemmatimonadaceae | 0.034 |
