# Supplementary material for: Revealing the Mechanism of Huazhi Rougan Granule in the Treatment of Nonalcoholic Fatty Liver Through Intestinal Flora Based on 16S rRNA, Metagenomic Sequencing and Network Pharmacology
Source: Front Pharmacol. 2022 Apr 26;13:875700. doi: 10.3389/fphar.2022.875700 (PMC9086680; doi:10.3389/fphar.2022.875700)
Supplement: Supplementary file 6 [file Table6.DOCX]

Additional file 6 Statistical results of sample sequencing data quality control of metagenomics

| Group/Index | Clean data base(bp) | Number of Reads | GC(%) | Q20(%) | Q30(%) |
| --- | --- | --- | --- | --- | --- |
| BC | 6412495060±110643541.90 | 15391950.89±270937.18 | 46.13±0.29 | 97.81±0.05 | 94.19±0.11 |
| MC | 6418819072.44±48089732.71 | 15133513.78±191289.63 | 46.99±0.13 | 97.93±0.12 | 94.46±0.24 |
| TL | 6755228518.44±109226370.13 | 16048189.22±232126.18 | 47.50±0.40 | 97.68±0.05 | 93.94±0.12 |
| TM | 6633565697.11±130715825.67 | 16130621.67±756918.83 | 47.20±0.26 | 97.96±0.17 | 94.36±0.46 |
| TH | 6842179110±140943836.73 | 19268743.56±393317.84 | 47.32±0.20 | 97.21±0.02 | 92.23±0.05 |
| PC | 6937753306.44±190054280.49 | 19584721.67±513596.75 | 47.69±0.35 | 97.24±0.05 | 92.32±0.12 |
